# Rates of convergence for regression with the graph poly-Laplacian

**DOI:** 10.1007/s43670-023-00075-5

**Published:** 2023-11-27

**Authors:** Nicolás García Trillos, Ryan Murray, Matthew Thorpe

**Affiliations:** 1 Department of Statistics, University of Wisconsin–Madison, Madison, WI 53706 USA; 2 Department of Mathematics, North Carolina State University, Raleigh, NC 27695 USA; 3 Department of Statistics, University of Warwick, Coventry, CV4 7AL UK

**Keywords:** Non-parametric regression on unknown domains, Supervised learning, Asymptotic consistency, Rates of convergence, PDEs on graphs, Nonlocal variational problems, 49J55, 49J45, 62G20, 35J20

## Abstract

In the (special) smoothing spline problem one considers a variational problem with a quadratic data fidelity penalty and Laplacian regularization. Higher order regularity can be obtained via replacing the Laplacian regulariser with a poly-Laplacian regulariser. The methodology is readily adapted to graphs and here we consider graph poly-Laplacian regularization in a fully supervised, non-parametric, noise corrupted, regression problem. In particular, given a dataset $\{x_i\}_{i=1}^n$ and a set of noisy labels $\{y_i\}_{i=1}^n\subset \mathbb {R}$ we let $u_n{:}\{x_i\}_{i=1}^n\rightarrow \mathbb {R}$ be the minimizer of an energy which consists of a data fidelity term and an appropriately scaled graph poly-Laplacian term. When $y_i = g(x_i)+\xi _i$, for iid noise $\xi _i$, and using the geometric random graph, we identify (with high probability) the rate of convergence of $u_n$ to *g* in the large data limit $n\rightarrow \infty$. Furthermore, our rate is close to the known rate of convergence in the usual smoothing spline model.

## Introduction

Given the applications to signal processing and computer science the smoothing spline problem has been attracting the interest of statisticians since the 1960s [1, 2]. The problem can be stated as [3]: given feature vectors $\{x_i\}_{i=1}^n\subset \Omega \subset \mathbb {R}^d$ and labels $\{y_i\}_{i=1}^n\subset \mathbb {R}$ minimize

1 $$\begin{aligned} \mathcal {E}_n^{(\textrm{spline})} (u) = \frac{1}{n} \sum _{i=1}^n |y_i - u(x_i)|^2 + \tau \Vert \nabla ^s u\Vert _{\textrm{L}^{2}(\Omega )}^2 \end{aligned}$$

over all $u\in \textrm{H}^{s}(\Omega )$, where $\textrm{H}^{s}(\Omega )$ is the Sobolev space with square integrable *s*th (weak) derivative on $\Omega$. Here, one tries to find an unknown function $u{:}\ \Omega \rightarrow \mathbb {R}$ that is trying to match the observed labels $\{y_i\}_{i=1}^n$ at $\{x_i\}_{i=1}^n$ (through the data fidelity term $\frac{1}{n} \sum _{i=1}^n |y_i - u(x_i)|^2$) whilst being smooth in the sense of $\textrm{H}^{s}$ regularization (by including the regularization term $\tau \Vert \nabla ^s u\Vert _{\textrm{L}^{2}(\Omega )}^2$). It is important to note that the regularity penalty is applied uniformly throughout the domain $\Omega$.

Recently, the spline methodology has found use in data science and machine learning as a candidate for semi-supervised or fully-supervised learning. Zhu et al. [4] introduced the following variational problem as a method for finding missing labels. They assumed that for every pair of feature vectors $x_i,x_j$ with $i,j\in \{1,\ldots ,n\}$ one has a measure of similarity $W_{i,j}$. Further assuming that there is no error in the observed labels $\{y_i\}_{i\in Z_n}$, where $Z_n\subsetneq \{1,\ldots ,n\}$, they proposed the variational problem: minimize

2 $$\begin{aligned} \mathcal {E}_n^{(\textrm{ZGL})}(u) = \sum _{i,j=1}^n W_{i,j} |u(x_i) - u(x_j)|^2 \end{aligned}$$

subject to $u(x_i) = y_i$ for all $i\in Z_n$ over $u{:}\ \{x_i\}_{i=1}^n\rightarrow \mathbb {R}$. The power of this method is that the regularization will be applied more strongly when the density of data is higher, at least when the weights $W_{i,j}$ are based on the proximity of points in the Euclidean sense. Indeed, in the $\varepsilon$-graph setting, the continuum, $n\rightarrow \infty$, limit of (2) is, up to a multiplicative constant,

3 $$\begin{aligned} \mathcal {E}_\infty (u) = \int _\Omega |\nabla u(x)|^2 \rho ^2(x) \, \textrm{d}x \end{aligned}$$

where $\rho$ is the density of the data. We see that where $\rho$ is large the minimizer of (3) should be smoother, and conversely when $\rho$ is smaller the minimizer can fluctuate more. This is typically desirable behaviour in classification tasks since the minimizer can be expected to be approximately constant within clusters and quickly transitioning outside of clusters where the density of data is assumed to be low.

Several types of convergence results connecting (2) to (3) exist in the literature. For instance pointwise convergence of the objective functionals was established in [5, 6]. Rates of convergence between constrained minimizers of (2) to constrained minimizers of (3) appeared in [7]. Further results concern the convergence of the Laplacian operator, without rates in [8–10] and with rates in [11–14], the game theoretic Laplacian in [15], the *p*-Laplacian in [16], and the $\infty$-Laplacian in [17]. However, none of these results consider *asymptotic consistency* in the sense that the minimizer of (2) converges to a “true function”. (It would be more accurate to describe the above results as *convergence* properties of the method.) To our knowledge we are the first to consider consistency in the graph-based setting.

When there is uncertainty in the observed labels, minimising (2) with constraints is not the natural model. Instead, as in (1), we can reformulate the Zhu et al. model in the fully supervised setting with *soft* labels by

4 $$\begin{aligned} \mathcal {E}_{n,\tau }^{(\textbf{y}_n)}(u) = \frac{1}{n} \sum _{i=1}^n |u(x_i) - y_i|^2 + \frac{\tau }{n^2\varepsilon ^2} \sum _{i,j=1}^n W_{i,j} |u(x_i) - u(x_j)|^2 \end{aligned}$$

where we include the correct scaling on the second term. The parameter $\tau$ controls the weighting between regularity and matching the data: formally, $\tau =0$ corresponds to the hard constrained problem. In some settings the large data limits for minimizers of (4) can be inferred from the *hard* constrained problem, see [7].

To complete the generalization of the Zhu et al. model to an analogue of the spline problem (1) we discuss higher order regularization. The Dirichlet energy $\mathcal {E}_n^{(\text {ZGL})}$ can be written in inner product form:

$$\begin{aligned} \mathcal {E}_n^{(\text {ZGL})}(u) = \langle \Delta _n u, u\rangle _{\textrm{L}^{2}(\mu _n)} \end{aligned}$$

where $\Delta _n$ is the graph Laplacian and $\textrm{L}^{2}(\mu _n)$ is the space of $\textrm{L}^{2}$ functions with respect to the empirical measure $\mu _n$ (defined in the following subsection). A natural method to introduce higher order regularity is to consider higher powers of the graph-Laplacian, in particular

5 $$\begin{aligned} \mathcal {E}_{n,\tau }^{(\textbf{y}_n)}(u) = \frac{1}{n} \sum _{i=1}^n (u(x_i) - y_i)^2 + \tau \langle \Delta _n^s u, u\rangle _{\textrm{L}^{2}(\mu _n)}, \end{aligned}$$

in which case (4) is the special case of (5) with $s=1$. In the context of graphs this model was introduced in [18], although the fractional Laplacian, including non-local and discrete versions, has been of interest in applied mathematics for much longer, see for example [19] and references therein. As observed in [4] and (in terms of uncertainty quantification) [20], (5) has an interpretation as a maximum a-posteriori (MAP) estimate for the Gaussian process regression method (also known as kriging). As an aside we mention works that have explored the connection between discrete and continuum problems in the Bayesian setting such as [21, 22] and [23], where the latter introduces Matérn priors on graphs and studies their continuum limits. In this paper we use the setting of [18] to recover a labeling function *g* as the MAP estimator given the data $\{(x_i,y_i)\}_{i=1}^n$ and using the graph poly-Laplacian to define a prior. In this Bayesian context, these works identify the higher-order regularizers as beneficial for fitting functions which are expected to have higher degree of regularity. Our focus will be on recovering the labels *g* *as well as* some of its higher order information at the data points $\{ x_i \}_{i=1}^n$ in the form of powers of the Laplacian of *g*.

Theoretical analysis of splines dates back to the 1960s and we refer to [3] for an overview of more historical works and only mention a few select (more recent) references here related to large data limits. Convergence in norm of special splines under various settings have been studied in [24–32] and pointwise convergence results in [33–37]. The optimal rates of convergence for special splines were established in [38] with rate $\Vert u_\tau ^*-g\Vert _{\textrm{L}^{2}(\Omega )}=O(n^{-\frac{s}{2s+d}})$ where $u_\tau ^*$ is the spline estimate minimizing (1). The result in [38] further established rates of convergence of derivatives, in particular showing $\Vert \nabla ^k u_\tau ^*-\nabla ^k g\Vert _{\textrm{L}^{2}(\Omega )}=O(n^{-\frac{s-k}{2\,s+d}})$. The general splines problem writes (1) in a more abstract framework. In particular, one seeks to find $u\in \mathcal {H}$, where $\mathcal {H}$ is a reproducing kernel Hilbert space that can be decomposed $\mathcal {H}= \mathcal {H}_0\oplus \mathcal {H}_1$, as the minimizer of

$$\begin{aligned} \mathcal {E}_n^{(\textrm{gen}\,\textrm{spline})} (u) = \frac{1}{n} \sum _{i=1}^n |y_i - L_iu|^2 + \tau \Vert P_1 u\Vert _{\mathcal {H}}^2 \end{aligned}$$

where $L_i\in \mathcal {H}^*$ (where $\mathcal {H}^*$ is the dual space of $\mathcal {H}$) and $P_1{:} \ \mathcal {H}\rightarrow \mathcal {H}_1$ is the orthogonal projection. The motivation for the model is that $\mathcal {H}_1$ is an infinite dimensional space where one wants to regularise, whilst $\mathcal {H}_0$ is a finite dimensional space which doesn’t need regularization. For an appropriate choice of $\mathcal {H}$ and $L_i$ one recovers the special smoothing spline problem (as a special case of the general smoothing spline problem). In particular, $\mathcal {H}_0$ is the space of polynomials of degree at most *s* and $\mathcal {H}_1$ is the subspace of $\textrm{H}^{s}$ with $\nabla ^k u=0$ for $k=0,\dots ,s-1$ and norm $\Vert \nabla ^s u\Vert _{\textrm{L}^{2}}$, see [39] for further details. The general smoothing spline problem has itself attracted attention with large data convergence in norm results appearing in [40–45] and weak convergence results in [39].

In this paper, using the model (5), we consider the problem of non-parametric regression of a noisy signal *g* observed at finitely many points that are randomly selected from an *unknown* probability distribution $\mu$ on the torus $\mathcal {T}$. More precisely, we assume we are given a set of feature vectors $\{x_i\}_{i=1}^n\subset \Omega :=\mathcal {T}$ and a set of associated noisy real valued labels $\{y_i\}_{i=1}^n$ satisfying

6 $$\begin{aligned} y_i = g(x_i)+ \xi _i, \end{aligned}$$

from which we wish to recover the true signal *g*, also known as the label function. The random variables $\xi _i$ are assumed to be mean zero, sub-Gaussian and independent. Our main results establish variance and bias estimates for the error of approximation of the signal *g* and of some of its higher order information by the solution *u* of a variational problem characterized by a graph PDE of the form

7 $$\begin{aligned} \tau \Delta _n^s u + u = y. \end{aligned}$$

Up to logarithms, we establish an $O( n^{-\frac{s}{d+4s}} )$ rate of convergence of minimizers of $\mathcal {E}_{n,\tau }^{(\textbf{y}_n)}$ in (5) to *g*. This is comparable to the $O(n^{-\frac{s}{d+2s}})$ rate of convergence in splines, see [38]. Projecting the samples onto the first *K* eigenvectors of the graph Laplacian gives a better rate of $O(n^{-\frac{s}{d+2s}})$ (the minimax rate) [46], however the optimal choice *K* depends on the $\textrm{H}^{s}$ norm of *g*, which will usually be unknown. See Remarks 9 and 10 for further details.

Methods such as kernel ridge regression are closely related to smoothing spline models. These methods use a data fidelity (on $\{x_i\}_{i=1}^n$) plus regularization on $\Omega$ (in particular incorporating $\Omega$’s geometry *explicitly*) to attempt to recover *g*. In that setting, the question of how to set regularization parameters was studied in [47, 48]. Some very recent works have focused on studying the “ridgeless case”, where one considers the limit as one sets the regularization parameter to zero, with both positive [49] and negative [50] results depending on the richness of the data. A related approach is to interpolate between data points using $\textrm{C}^{m}$ penalization [51–53] or Sobolev penalization [54–56].

There are connections between the Gaussian process regression (kriging) method approach that we take here and the generalized lasso model (which includes the lasso, the fused lasso, trend filtering, and the more closely related to our work: graph fused lasso, graph trend filtering, and Kronecker trend filtering), see for example [57]. Both Gaussian process regression and generalized lasso attempt to recover an unknown function *g* from noisy observations of the form (6) in the fully supervised setting (i.e. for each feature vector $x_i$ we have an observation $y_i$). However, in the lasso models the function *g* is assumed to be linear, i.e. $g(x) = \beta \cdot x$ where $\beta$ is an unknown vector which parametrises *g*. The fused lasso, on the other hand, uses a total variation regularization in place of the graph poly-Laplacian considered here. In grid graphs this has been considered in [58–60] where the estimator is shown to be minimax (the estimator performs best amongst all other estimators in the worst case). Further results have considered chain graphs [61], and *k*-NN and $\varepsilon$-connected graphs [62] (the $\varepsilon$-connected graph setting is also the setting of this paper). In particular, the $\text {L}^2$ convergence rate of the fused lasso on an $\varepsilon$-connected graph is (ignoring logarithms) $O(n^{-\frac{1}{d}})$ which at least in some settings is the minimax rate [62]. Up to logarithms, and assuming a sufficiently smooth signal *g*, our basic $\text {L}^2$ convergence rates for the approximation of *g* coincide with these rates. This is also approximately the $\text {L}^\infty$ convergence rate given in [63] for the case $s=1$ in (7), which is the minimax $\text {L}^\infty$ rate given in [64]. At this point we would like to remark that our variance estimates are meaningful and converge to zero with growing *n* even if the regularization parameter $\tau$ is not scaled down to zero. Our results characterize precisely the continuum limit of the solutions to the graph PDE (7), and are of relevance in case one were interested, not only on denoising, but also in enforcing additional regularization. We also remark that in our results we provide additional information about the convergence towards *g*, by giving convergence rates for higher-order derivatives.

Other approaches for high order regularization that do not consider Gaussian priors use instead a non-linear *p*-Laplacian operator for large enough *p* defined by

$$\begin{aligned} \Delta _n^{(p)} u(x_i) = \frac{1}{n\varepsilon ^p} \sum _{j=1}^n W_{ij} (u(x_i)-u(x_j)) |u(x_i)-u(x_j)|^{p-2} \end{aligned}$$

(which is consistent with the definition in (8) for $p=2$ and written $\Delta _n:=\Delta _n^{(2)}$). In the graph setting, results like those in [65] establish that solutions of a *p*-Dirichlet regularised problem converge with rate $n^{-\frac{1}{4}}$ to the solution of an analogue continuum non-local variational problem; although the setting differs from ours as we scale the connectivity of our graph to obtain a local limit whilst in [65] the connectivity of the graph remains fixed and the limit is to a nonlocal variational problem. Naturally, the advantage of the framework in [65] is that the dimension of the space essentially plays no role in the analysis (depending on the precise edge model one uses) and therefore it is enough to consider the problem in 1D (as the authors do). On the other hand, by not scaling down the connectivity threshold it is not possible to recover the local geometry. The same authors, in the same setting, show a rate of convergence for the associated gradient flow [66]. As was mentioned earlier when discussing generalized Lasso models (in particular in graph trend filtering), total variation is another tool used to regularise regression and classification problems. This has motivated theoretical works like [67] which study the convergence of graph total variation to a continuum weighted total variation (the same paper proposed a topology to study the convergence that didn’t require regularity—in particular pointwise evaluation—of the continuum function). Total variation functionals are also widely used for clustering and segmentation such as in graph cut methods, for example ratio or Cheeger cuts [68, 69], graph modularity clustering [70, 71], and Ginzburg–Landau segmentation [72–74].

Since we have observations for all feature vectors our problem is in the fully-supervised setting. The semi-supervised setting with Laplacian regularization (closely related to (7) with $s=1$ but with hard constraints as opposed to having a penalty term) has been considered in [7] which show an “ill-posedness result” (the labels disappear in the large data limit) if the number of labelled points scales below a critical threshold, and a “well-posedness result” (the labels remain in the continuum problem) when the number of labels scales linearly with *n*. Using graph *p*-Laplacian regularization with finite labels the authors in [15, 16] show that whether the variational problem is asymptotically well-posed depends on the choice of *p* and how the graph is scaled. For the fractional graph Laplacian with finite labels, it is shown in [18] that the problem is ill-posed if $s\le \frac{d}{2}$ or the length scale on the graph is sufficiently large and conjectured that this is sharp.

We also mention that other approaches to regression on unknown manifolds include [75], where local tangent planes around points are carefully constructed to apply regression methods in the more classical functional data setting. Our approach is markedly different as it does not rely on the construction of *extrinsic* geometric objects. In particular, once a proximity graph is defined on the data cloud, all regularisers and the resulting PDEs become *intrinsic* to the graph.

Here we have reviewed a wide range of models which incorporate higher-order regularity. From a high-level, the inclusion of higher-order regularity terms will lead to improved fitting when the underlying source of labels has more regular dependence on the features, and this is quantified by concrete consistency results. Furthermore, higher-order regularizers have proved useful in smoothly propagating labels in semi-supervised and Bayesian learning settings. This work adds to this literature in the context of higher-order Laplacian regularization.

In the remainder of this section we define the graph and continuum operators that are analysed in the paper, and then state our main results.

### Discrete operators

We begin by stating our basic assumptions on the data, and the graph that we use to model it:

1. **Assumptions on the domain** $\Omega$ **:** $\Omega$ is a *d*-dimensional torus.

The assumption that $\Omega$ is a torus is largely to simplify our arguments; we expect the same result to hold on manifolds without boundaries. For sets with boundaries (either in the manifold setting or when $\Omega$ is an open subset of $\mathbb {R}^d$) we do not expect the same results. This is because the rate of convergence of the graph Laplacian to its continuum limit deteriorates near the boundary, see for example [7, Theorem 3.2]. We leave the question of rates of convergence for regression with the graph poly-Laplacian open in this setting.

(A2) **Assumptions on the feature vectors** $x_i$ **:** $x_i{\mathop {\sim }\limits ^{\textrm{iid}}}\mu$ where $\mu \in \mathcal {P}(\Omega )$, where $\mathcal {P}(\Omega )$ is the set of probability measures on $\Omega$;
(A3) **Assumption on the density of** $\mu$ **:** $\mu$ has a density $\rho \in \text {C}^\infty (\Omega )$ that is bounded from above and below by positive constants, i.e. $0<\rho _{\min }:= \min _{x\in \Omega } \rho (x) \le \max _{x\in \Omega } \rho (x) =: \rho _{\max } <+\infty$.
(A4) **Assumptions on the graph constructed using the data** $\{x_i\}$ **:** $G_n:= (\Omega _n,W_n)$ where $\Omega _n=\{x_i\}_{i=1}^n$ are the nodes and $W_n=(W_{i,j})_{i,j=1}^n$ are the edge weights defined by $W_{i,j} = \eta _{\varepsilon }(|x_i-x_j|)$ for $i\ne j$ and $W_{i,i}=0$. Here $\eta _{\varepsilon } = \frac{1}{\varepsilon ^d} \eta (\cdot /\varepsilon )$ and where $\eta :[0,+\infty )\rightarrow [0,+\infty )$ is assumed to satisfy:
     (a) $\eta (t)>\frac{1}{2}$ for all $t\le \frac{1}{2}$ and $\eta (t)=0$ for all $t\ge 1$;
     (b) $$\eta $$ is decreasing.
(A5) **Assumptions on the labelled data :** for each $i\in \mathbb {N}$, $y_i= g(x_i)+\xi _i$, for $g\in \textrm{H}^{2\,s+1+\frac{d}{2}}(\Omega )$. and $\xi _i\in \mathbb {R}$ are independent and identically distributed (iid), sub-Gaussian centred noise (where sub-Gaussian by definition means there exists $C>c>0$ such that $\mathbb {P}\left( |\xi _j|>t\right) \le Ce^{-ct^2}$ for all $t\ge 0$).

Our model assumes noise in the labels, but not in the feature vectors. An interesting further question would be what happens if feature vectors are also noisy. This question has been partially studied in the context of adversarial noise (where the adversary perturbs the feature vectors) in the semi-supervised setting; the results of [76] show that if the adversary cannot move feature vectors more than some maximum distance then the method is still asymptotically consistent.

#### Remark 1

1. The assumption that $g\in \textrm{H}^{2s+1+\frac{d}{2}}$ is because we show $\Vert u_\tau ^*\Vert _{\textrm{H}^{k}(\Omega )}\lesssim \Vert g\Vert _{\textrm{H}^{k}(\Omega )}$, where $u_\tau ^*$ is the noiseless solution in the continuum setting (i.e. solves $\tau \Delta ^s_\rho u + u = g$ where $\Delta _\rho$ is the continuum limit of the graph Laplacian, see (12) and compare to (7)). By Morrey’s inequality we have that $u_\tau ^*\in \textrm{C}^{2s+1}(\Omega )$, and hence $u_\tau ^*$ solves the above equation in a strong sense (see Lemma 2.14 for details).
2. The assumptions on $\eta$ are technical in nature and are imposed to facilitate some very concrete steps in our analysis. Assumption (A4)(b) is slightly stronger than what is typically required in related papers, and will only be used to simplify our computations in, for example, Lemma 2.3.

The graph Laplacian $\Delta _n$ plays an important role in the regularization and is defined as follows:

8 $$\begin{aligned} \Delta _n := \frac{2}{n\varepsilon ^2}(D_n-W_n), \qquad D_n=(D_{i,j})_{i,j=1}^n \text { diagonal matrix with } D_{i,i} = \sum _{k=1}^n W_{i,k}. \end{aligned}$$

Here we have chosen what is called the *unnormalized* graph Laplacian.

Throughout the paper we will denote the empirical measure $\mu _n:= \frac{1}{n} \sum _{i=1}^n \delta _{x_i}$. We will define an inner product with respect to a (usually probability) measure $\nu$ by

$$\begin{aligned} \langle u,v \rangle _{\text {L}^2(\nu )}:= \int _\Omega u(x) v(x) \, \textrm{d}\nu (x) \qquad \text {for } u,v \text { measurable w.r.t. } \nu , \end{aligned}$$

and the associated $\text {L}^2$ norm by $\Vert u\Vert _{\text {L}^2(\nu )} = \sqrt{\langle u,u\rangle _{\text {L}^2(\nu )}}$. When $\nu = \mu _n$ then the norm can be written $\Vert u\Vert _{\text {L}^2(\mu _n)} = \sqrt{\frac{1}{n} \sum _{i=1}^n u(x_i)^2}$.

There is a small abuse in notation in how we define $\Delta _n$ since we will also write $\Delta _n{:} \ \text {L}^2(\mu _n) \rightarrow \text {L}^2(\mu _n)$; in this case we associate $u_n\in \text {L}^2(\mu _n)$ with its vector representation $(u_n(x_1),\ldots , u_n(x_n))^\top$. With a secondary abuse of notation we can apply $\Delta _n$ to a continuous function on a continuum domain. That is, if $u\in \textrm{C}^{0}(\Omega )$ then we can interpret $\Delta _n u = \Delta _n (u\lfloor _{\Omega _n})$ as the graph Laplacian applied to the restriction of *u* onto the data points $\Omega _n=\{x_i\}_{i=1}^n \subset \Omega$.

We can define the graph derivative by $\nabla _n u(x_i,x_j) = \frac{1}{\varepsilon } \sqrt{W_{ij}} (u_j-u_i)$ which is an anti-symmetric divergence (i.e. satisfies $\nabla _n u(x_i,x_j)=-\nabla _n u(x_j,x_i)$). Using the norm

$$\begin{aligned} \langle U,V\rangle _{\textrm{L}^{2}(\mu _n\otimes \mu _n)} = \frac{1}{n^2} \sum _{i,j=1}^n U(x_i,x_j) V(x_i,x_j) \end{aligned}$$

on the set of anti-symmetric divergences $U{:} \ \{x_i\}_{i=1}^n \times \{x_i\}_{i=1}^n \rightarrow \mathbb {R}$ we can define the graph divergence to be the negative adjoint of the graph derivative, i.e. $\textrm{div}_n U (x_i) = \frac{2}{n\varepsilon } \sum _{j=1}^n \sqrt{W_{ij}} U(x_i,x_j)$. The normalization in the graph derivative is chosen so that the graph Laplacian can be defined as the negative of the graph divergence applied to the graph derivative: $\Delta _n = -\textrm{div}_n \circ \nabla _n$.

Given $\textbf{a}_n = (a_1,\ldots , a_n),$ with $a_i \in {\mathbb {R}}$, we let

9 $$\begin{aligned} \mathcal {E}_{n,\tau }^{(\textbf{a}_n)}(u_n)= \frac{1}{n} \sum _{i=1}^n |u_n(x_i) - a_i|^2 + \tau R_n^{(s)}(u_n), \end{aligned}$$

where the regularization is given by

10 $$\begin{aligned} R_n^{(s)}(u_n) = \langle \Delta _n^s u_n,u_n\rangle _{\text {L}^2(\mu _n)}, \end{aligned}$$

and here *s* is a positive integer with $\Delta _n^s$ the *s*-th power of the matrix. We will mostly be concerned with the situation where $\textbf{a}_n = \textbf{y}_n = (y_1,\ldots , y_n)$, which gives the energy

11 $$\begin{aligned} \mathcal {E}_{n,\tau }^{(\textbf{y}_n)}(u_n) = \frac{1}{n} \sum _{i=1}^n |u_n(x_i) - y_i|^2 + \tau R_n^{(s)}(u_n) \end{aligned}$$

We will define $u_{n,\tau }^*$ to be the minimizer of (11). Note that when $s=1$,

$$\begin{aligned} R_n^{(1)}(u_n) = \frac{1}{n^2\varepsilon ^{2}} \sum _{i,j=1}^n W_{i,j} |u_n(x_i) - u_n(x_j)|^2 \end{aligned}$$

and the regularization functional is the graph Dirichlet energy. We define $R_n^{(s)}$ for non-integer powers via the eigenvector–eigenvalue expansion (however our results consider only integer powers). That is, we let $(\lambda _i^{(n)},q_i^{(n)})$ be eigenpairs of $\Delta _n$ then, since $\{q_i^{(n)}\}_{i=1}^n$ form an orthonormal basis of $\text {L}^2(\mu _n)$, we can write (for any $s\in \mathbb {R}$)

$$\begin{aligned} R_n^{(s)}(u_n) = \sum _{i=1}^n (\lambda _i^{(n)})^s \langle u_n,q_i^{(n)}\rangle _{\text {L}^2(\mu _n)}^2. \end{aligned}$$

### Continuum operators

We now define the appropriate continuum operators and variational formulations. It is well-known that as $n \rightarrow \infty$, the operator $\Delta _n$ converges to a continuum limit $\Delta _\rho$ [7, 9–13, 15], where $\Delta _\rho$ is the differential operator defined by

12 $$\begin{aligned} \Delta _\rho \phi := -\frac{\sigma _\eta }{\rho } \textrm{div}(\rho ^2\nabla \phi ) \end{aligned}$$

and $\sigma _\eta$ is the constant defined by

13 $$\begin{aligned} \sigma _\eta := \int _{\mathbb {R}^d} \eta (|h|) |h_1|^2 \, \textrm{d}h. \end{aligned}$$

For $\tau >0$ fixed, the continuum objective functional is defined by

14 $$\begin{aligned} \mathcal {E}_{\infty ,\tau }^{(g)}(u) = \int _\Omega |u(x) - g(x)|^2 \rho (x)\, \textrm{d}x + \tau R_\infty ^{(s)}(u) \end{aligned}$$

where

15 $$\begin{aligned} R_\infty ^{(s)}(u) = \langle \Delta _\rho ^s u,u\rangle _{\text {L}^2(\mu )}. \end{aligned}$$

We will define $u_\tau ^*$ to be the minimizer of (14). Again, we observe that when $s=1$ the regularization functional,

$$\begin{aligned} R_\infty ^{(1)}(u) = \sigma _\eta \int _\Omega |\nabla u(x)|^2 \rho ^2(x) \, \textrm{d}x, \end{aligned}$$

is a weighted Dirichlet energy.

We remark that, by the fact that $\rho$ is bounded from below, we may integrate by parts to obtain

$$\begin{aligned} cR_\infty ^{(s)}(u) \le \int _\Omega | \nabla ^s u(x)|^2\,\textrm{d}x \le C R_\infty ^{(s)}(u) \end{aligned}$$

for some constants $C>c>0$.

We can also define $R_\infty ^{(s)}$ for non-integer powers analogously to the discrete case. More concretely, by the spectral theorem and the fact that $\Omega$ is compact, if we let $(\lambda _i,q_i)$ be eigenpairs of $\Delta _\rho$ then $\{q_i\}_{i=1}^\infty$ form an orthonormal basis of $\text {L}^2(\mu )$. In turn we can define

$$\begin{aligned} R_\infty ^{(s)}(u) = \sum _{i=1}^\infty \lambda _i^s \langle u,q_i\rangle _{\text {L}^2(\mu )}^2 \end{aligned}$$

which is well-defined for any $s\in \mathbb {R}$.

### Main results

Our results are to bound the bias and variance of the estimator $u_{n,\tau }^*$, defined as the minimizer of $\mathcal {E}_{n,\tau }^{(\textbf{y}_n)}$. Following the terminology of [47] we define the variance of the estimator by

$$\begin{aligned} \Vert u_{n,\tau }^* - u^*_\tau \lfloor _{\Omega _n} \Vert _{\text {L}^2(\mu _n)} \end{aligned}$$

where $u^*_\tau$ is the minimizer of $\mathcal {E}_{\infty ,\tau }^{(g)}$ and $\lfloor _{\Omega _n}$ is the restriction to $\Omega _n$, and the bias is defined to be

$$\begin{aligned} \Vert u^*_\tau - g \Vert _{\text {L}^2(\mu )}. \end{aligned}$$

The main results are the following.

#### $\textrm{L}^{2}$ variance estimates

We state the $\textrm{L}^{2}$ variance estimates in the following theorem.

##### Theorem 1.1

(Variance Estimates) Let Assumptions (A1)–(A5) hold and $s\in \mathbb {N}$. We define $\mathcal {E}_{n,\tau }^{(\textbf{y}_n)}$ by (11) and $\mathcal {E}_{\infty ,\tau }^{(g)}$ by (14) where $R_n^{(s)}$ is defined by (10), $R_\infty ^{(s)}$ by (15), $\Delta _n$ by (8) and $\Delta _\rho$ by (12). Let $u_{n,\tau }^*$ be the minimizer of $\mathcal {E}_{n,\tau }^{(\textbf{y}_n)}$ and $u^*_\tau$ be the minimizer of $\mathcal {E}_{\infty ,\tau }^{(g)}$. Then, for all $\alpha >1$, there exists $\varepsilon _0>0$, $\tau _0>0$, $C>c>0$ such that for all $\varepsilon ,n$ satisfying

16 $$\begin{aligned} \varepsilon _0 \ge \varepsilon \ge C\root d \of {\frac{\log (n)}{n}} \end{aligned}$$

and $\tau \in (0,\tau _0)$ we have

$$\begin{aligned} \Vert u_{n,\tau }^* - u^*_\tau \lfloor _{\Omega _n} \Vert _{\text {L}^2(\mu _n)}&\le C\left( \sqrt{\frac{\log (n)}{n\varepsilon ^{d}}} + \frac{\varepsilon ^{2s}}{\tau } + \tau \varepsilon \right) \end{aligned}$$

with probability at least $1-C\left( n^{-\alpha } + n e^{-cn\varepsilon ^{d+4s}}\right)$.

Let $\textbf{g}_n = (g(x_1),\ldots , g(x_n))$, and let $u_{n,\tau }^{g*}$ be the minimizer of the “noiseless” energy $\mathcal {E}_{n,\tau }^{(\textbf{g}_n)}$. The proof of Theorem 1.1 is divided into two steps: in the first we compare $u^*_{n,\tau }$ and $u^{g*}_{n,\tau }$ (corresponding to averaging in *y*) which gives a quantitative bound on the effect of the noise; in the second part we compare $u^{g*}_{n,\tau }$ with $u^*_\tau$ (which corresponds to averaging in *x*). We do this in Sects. 2.1 and 2.2 respectively.

##### Remark 2

We notice that the estimates are meaningful for fixed $\tau$ when *n* goes to infinity, i.e. $\tau$ is not required to become smaller with growing *n*.

##### Remark 3

In addition to the bound in $\text {L}^2(\mu _n)$ between $u_{n,\tau }^*$ and $u_\tau ^*$ we are able to show a bound between the Laplacians $\Delta _n^{\frac{s}{2}} u_{n,\tau }^*$ and $\Delta _\rho ^{\frac{s}{2}} u^*_\tau \lfloor _{\Omega _n}$ when *s* is even. More precisely, our results show,

$$\begin{aligned} \left\| \Delta _n^{\frac{s}{2}} u_{n,\tau }^* - \Delta _\rho ^{\frac{s}{2}} u_\tau ^*\lfloor _{\Omega _n} \right\| _{\text {L}^2(\mu _n)} \le C \left( \sqrt{\frac{\log (n)}{n\varepsilon ^d\tau }} + \frac{\varepsilon ^s}{\tau } + \varepsilon \right) \end{aligned}$$

with the same probability as in Theorem 1.1. This inequality likely generalizes to odd *s*, but to prove it using the methods in this paper we would require a pointwise convergence result for the graph derivative which is beyond the scope of the paper.

##### Remark 4

We offer a comparison with the estimates in [65] (although note that a direct comparison is not possible as we scale $\varepsilon \rightarrow 0$ whilst [65] work in the setting where $\varepsilon >0$ is fixed). If, as in [65], we fix $\tau >0$, and therefore absorb it into our constants, and choose $s=1$ then the error bound simplifies to

$$\begin{aligned} \Vert u_{n,\tau }^* - u^*_\tau \lfloor _{\Omega _n} \Vert _{\text {L}^2(\mu _n)} \le C\left( \sqrt{\frac{\log (n)}{n\varepsilon ^d}} + \varepsilon \right) . \end{aligned}$$

Unfortunately, optimising over $\varepsilon$ implies a scaling in $\varepsilon =\varepsilon _n$ of

$$\begin{aligned} \varepsilon _n \sim \left( \frac{\log (n)}{n}\right) ^{\frac{1}{d+2}} \end{aligned}$$

which is outside of the conditions of Theorem 1.1 as (16) does not hold (one needs $n\varepsilon ^{d+4}\gg \log (n)$ in order to get a high probability bound). Instead we choose $\varepsilon _n\sim \left( \frac{\log (n)}{n}\right) ^{\frac{1}{d+4}}$. With this choice the error scales as

$$\begin{aligned} \Vert u_{n,\tau }^* - u^*_\tau \lfloor _{\Omega _n} \Vert _{\text {L}^2(\mu _n)} \lesssim \left( \frac{\log (n)}{n} \right) ^{\frac{1}{d+4}}. \end{aligned}$$

The results in [65] show that for the *p*-Laplacian regularized problem a rate of convergence $n^{-\frac{1}{4}}$ when $d=1$, $s=1$ and $\varepsilon >0$ is fixed independently of *n*, compared to our rate of convergence of $n^{-\frac{1}{5}}$ (up to logarithms).

#### $\textrm{L}^{2}$ bias estimates

We have the following $\textrm{L}^{2}$ bias estimate.

##### Theorem 1.2

(Bias Estimates) Let Assumptions (A1), (A3) hold and $\tau >0$, $s\ge 1$ and $g\in \text {H}^s(\Omega )$. We define $\mathcal {E}_{\infty ,\tau }^{(g)}$ by (14) where $R_\infty ^{(s)}$ is defined by (15) and $\Delta _\rho$ by (12). Let $u_\tau ^*$ be the minimizer of $\mathcal {E}_{\infty ,\tau }^{(g)}$, then

$$\begin{aligned} \Vert u_\tau ^* - g \Vert _{\text {L}^2(\mu )} \le \tau \Vert \Delta _\rho ^s g\Vert _{\text {L}^2(\mu )}. \end{aligned}$$

The theorem is proved in Sect. 3.

##### Remark 5

We are also able to show that, for *s* even,

$$\begin{aligned} \left\| \Delta _\rho ^{\frac{s}{2}} (g-u_\tau ^*) \right\| _{\text {L}^2(\mu )} \le \sqrt{\frac{\tau }{2}} \left\| \Delta _\rho ^s g\right\| _{\text {L}^2(\mu )}. \end{aligned}$$

#### $\textrm{L}^{2}$ error estimates

The results from Sects. 1.3.1 and 1.3.2 can be combined to bound the error between $u_{n,\tau }^*$ and *g*.

##### Theorem 1.3

Let Assumptions (A1)–(A5) hold and let $s\in \mathbb {N}$. We define $\mathcal {E}_{n,\tau }^{(\textbf{y}_n)}$ by (11) where $R_n^{(s)}$ is defined by (10) and $\Delta _n$ by (8). Let $u_{n,\tau }^*$ be the minimizer of $\mathcal {E}_{n,\tau }^{(\textbf{y}_n)}$. Then for every $\alpha >1$ there exists $\varepsilon _0>0$, $C>c>0$ such that for all $\varepsilon , n$ satisfying (16) and $\tau \in (0,\tau _0)$ we have

$$\begin{aligned} \Vert u_{n,\tau }^* - g\lfloor _{\Omega _n} \Vert _{\text {L}^2(\mu _n)} \le C\left( \left( \frac{\log (n)}{n}\right) ^{1/4} + \sqrt{\frac{\log (n)}{n\varepsilon ^d}} + \frac{\varepsilon ^{2s}}{\tau } + \tau \right) \end{aligned}$$

with probability at least $1- C \left( n^{-\alpha } + ne^{-cn\varepsilon ^{d+4\,s}} \right)$.

##### Proof

By the triangle inequality and Theorem 1.2,

17 $$\begin{aligned} \Vert u_{n,\tau }^* - g\lfloor _{\Omega _n} \Vert _{\text {L}^2(\mu _n)}&\le \Vert u_{n,\tau }^* - u^*_\tau \lfloor _{\Omega _n} \Vert _{\text {L}^2(\mu _n)} + \Vert u^*_\tau \lfloor _{\Omega _n} - g\lfloor _{\Omega _n} \Vert _{\text {L}^2(\mu _n)} \nonumber \\&\le C\left( \sqrt{\frac{\log (n)}{n\varepsilon ^d}} + \frac{\varepsilon ^{2s}}{\tau } + \varepsilon \tau \right) + \Vert u^*_\tau \lfloor _{\Omega _n} - g\lfloor _{\Omega _n} \Vert _{\text {L}^2(\mu _n)} \end{aligned}$$

with probability at least $1-C(n^{-\alpha } + ne^{-cn\varepsilon ^{d+4s}})$.

To simplify notation, let $\omega _i = (u_\tau ^*(x_i) - g(x_i))^2$ then

$$\begin{aligned} \Vert u^*_\tau \lfloor _{\Omega _n} - g\lfloor _{\Omega _n} \Vert _{\text {L}^2(\mu _n)}&= \frac{1}{n} \sum _{i=1}^n (u^*_\tau (x_i) - g(x_i))^2 = \frac{1}{n} \sum _{i=1}^n \omega _i. \end{aligned}$$

We note that $\mathbb {E}[\omega _i] = \mathbb {E}[(u_\tau ^*(X)-g(X))^2] = \Vert u^*_\tau -g\Vert _{\text {L}^2(\mu )}^2$ and

$$\begin{aligned} 0 \le \alpha _i \le \sup _{x\in \Omega } \left( u_\tau ^*(x) - g(x)\right) ^2 \le 2\left( \Vert u_\tau ^*\Vert _{\textrm{L}^{\infty }}^2 + \Vert g\Vert _{\textrm{L}^{\infty }}^2\right) \le M \end{aligned}$$

for all $\tau <\tau _0$ by Lemma 2.14 and Assumption (A5). In particular, *M* is independent of *n* and $\tau$. Hoeffding’s inequality: for all $\zeta >0$,

$$\begin{aligned} \mathbb {P}\left( \frac{1}{n}\sum _{i=1}^n \alpha _i - \frac{1}{n}\sum _{i=1}^n \mathbb {E}[\alpha _i] \ge \zeta \right) \le \exp \left( -\frac{2n\zeta ^2}{M^2} \right) , \end{aligned}$$

with Theorem 1.1 implies, with probability at least $1-e^{-cn\zeta ^2}$,

$$\begin{aligned} \Vert u^*_\tau - g\lfloor _{\Omega _n} \Vert _{\text {L}^2(\mu _n)}^2 \le \Vert u_\tau ^*-g\Vert _{\text {L}^2(\mu )}^2 + \zeta \le \tau ^2\Vert \Delta _\rho ^s g\Vert _{\textrm{L}^{2}(\mu )}^2 + \zeta . \end{aligned}$$

We choose $\zeta ^2 = \frac{\alpha \log (n)}{cn}$ so $\Vert u^*_\tau - g\lfloor _{\Omega _n} \Vert _{\text {L}^2(\mu _n)}^2 \le \tau ^2\Vert \Delta _\rho ^s g\Vert _{\textrm{L}^{2}(\mu )}^2 + C\sqrt{\frac{\log (n)}{n}}$ with probability at least $1-n^{-\alpha }$. Substituting into (17) we have

$$\begin{aligned} \Vert u_{n,\tau }^* - g\lfloor _{\Omega _n} \Vert _{\text {L}^2(\mu _n)} \le C\left( \sqrt{\frac{\log (n)}{n\varepsilon ^d}} + \frac{\varepsilon ^{2s}}{\tau } + \varepsilon \tau + \tau \Vert \Delta _\rho ^s g\Vert _{\textrm{L}^{2}(\mu )} + \left( \frac{\log (n)}{n}\right) ^{1/4} \right) \end{aligned}$$

with probability at least $1-C(n^{-\alpha } + ne^{-cn\varepsilon ^{d+4s}})$. $\square$

##### Remark 6

In the above proof we could have obtained tighter estimates if we had used Bernstein’s inequality instead of Hoeffding’s inequality, since the variance of the random variables $(u_\tau ^*(x_i) - g(x_i) )^2$ can be easily proved to be of order $\tau ^2$. However, as we will see below, the loose estimates provided by Hoeffding’s inequality are of strictly smaller order than the errors that come from Theorem 1.1 and thus it is sufficient for our purposes to use these simpler bounds.

##### Remark 7

Combining Remarks 3 and 5 and using a similar strategy as the one used to obtain the estimates in Corollary 1.3 we can also show, for even *s*,

$$\begin{aligned} \left\| \Delta _n^{\frac{s}{2}} u_{n,\tau }^* - \Delta _\rho ^{\frac{s}{2}}g\lfloor _{\Omega _n} \right\| _{\text {L}^2(\mu _n)} \le C\left( \left( \frac{\log (n)}{n}\right) ^{1/4} + \sqrt{\frac{\log (n)}{\tau n\varepsilon ^d}} + \frac{\varepsilon ^{s}}{\tau } + \varepsilon + \sqrt{\tau } \right) \end{aligned}$$

with probability at least $1-C\left( n^{-\alpha } + ne^{-cn\varepsilon ^{d+4s}}\right)$.

##### Remark 8

When $s=1$ the error simplifies to

$$\begin{aligned} \Vert u_{n,\tau }^* - g\lfloor _{\Omega _n} \Vert _{\text {L}^2(\mu _n)} \le C\left( \left( \frac{\log (n)}{n}\right) ^{1/4} + \sqrt{\frac{\log (n)}{n\varepsilon ^d}} + \frac{\varepsilon ^{2}}{\tau } + \tau \right) \end{aligned}$$

with probability at least $1-C\left( n^{-\alpha } + ne^{-cn\varepsilon ^{d+4}}\right)$. Choosing $\tau$ optimally with respect to $\varepsilon$ implies $\tau =\varepsilon$ and

$$\begin{aligned} \Vert u_{n,\tau }^* - g\lfloor _{\Omega _n} \Vert _{\text {L}^2(\mu _n)} \le C\left( \left( \frac{\log (n)}{n}\right) ^{1/4} + \sqrt{\frac{\log (n)}{n\varepsilon ^d}} + \varepsilon \right) . \end{aligned}$$

The optimal choice of $\varepsilon$ is $\varepsilon _n = \left( \frac{\log (n)}{n}\right) ^{\frac{1}{d+2}}$, which (as in Remark 4) is outside the admissible scaling of $\varepsilon _n$, so we choose $\varepsilon _n\sim \left( \frac{\log (n)}{n}\right) ^{\frac{1}{d+4}}$. In this regime the optimal error is then

$$\begin{aligned} \Vert u_{n,\tau }^* - g\lfloor _{\Omega _n} \Vert _{\text {L}^2(\mu _n)} \le C\left( \frac{\log (n)}{n} \right) ^{\frac{1}{d+4}}, \end{aligned}$$

as the term $\left( \frac{\log (n)}{n}\right) ^{1/4}$ is always of smaller order. Notice that the above error rate is approximately the minimax rate achieved for the total variation regularised problem which, in certain cases, is up to logarithms scaling as $n^{-\frac{1}{d}}$ [62], comparable to the $\text {L}^\infty$ minimax rates and convergence of spline smoothing obtained in [38, 63, 64], which are approximately $n^{-\frac{1}{d+2}}$. This also coincides with the semi-supervised rate of convergence given in [7] when the number of labeled data is linear in *n*.

##### Remark 9

For $s\in \mathbb {N}$ we can choose $\tau = \varepsilon ^s$ so that the error simplifies to

$$\begin{aligned} \Vert u_{n,\tau }^* - g\lfloor _{\Omega _n} \Vert _{\text {L}^2(\mu _n)} \le C\left( \left( \frac{\log (n)}{n}\right) ^{1/4} + \sqrt{\frac{\log (n)}{n\varepsilon ^d}} + \varepsilon ^s \right) \end{aligned}$$

with probability at least $1-C(n^{-\alpha } + ne^{-cn\varepsilon ^{d+4s}})$. If we choose $\varepsilon _n \sim \left( \frac{M\log (n)}{n}\right) ^{\frac{1}{d+4s}}$, then the error scales as

$$\begin{aligned} \Vert u_{n,\tau }^* - g\lfloor _{\Omega _n} \Vert _{\text {L}^2(\mu _n)} \le C\left( \left( \frac{\log (n)}{n}\right) ^{1/4} + \left( \frac{\log (n)}{n}\right) ^{\frac{s}{d+4s}} \right) \le C\left( \frac{\log (n)}{n}\right) ^{\frac{s}{d+4s}} \end{aligned}$$

(where *C* depends on *M*) with probability at least $1-C\left( n^{-\alpha }+ n^{1-cM}\right)$, choosing $M=\frac{1+\alpha }{c}$ we have that the bound holds with probability at least $1-Cn^{-\alpha }$. This is close to the spline error rate, which, up to logarithms, scales as $n^{-\frac{s}{d+2s}}$; see [38].

##### Remark 10

The minimax rates for estimating *g* from noisy samples (6) is $n^{-\frac{s}{2s+d}}$ when $g\in \text {H}^s$ (the rate achieved by splines). In the graph setting the minimax rate can be obtained by projecting the samples onto the first $K=K(\Vert g\Vert _{\text {H}^s})$ eigenvectors of the graph Laplacian [46]. Whilst this has the advantage of better rates, one must have an a-priori estimate in the $\text {H}^s$ norm of *g* in order to know *K*.

### Outline

The rest of the paper is organized as follows. In Sect. 2 we obtain the $\text {L}^2$ variance estimates discussed in Sect. 1.3.1. In Sect. 3 we consider the bias of the estimation procedure given in Sect. 1.3.2.

## $\textrm{L}^{2}$ variance estimates

In this section we prove the variance estimates stated precisely in Theorem 1.1. We split the proof into two main steps. First, we compare the solution $u_{n,\tau }^*$ with a discrete noiseless function $u_{n,\tau }^{g*}$. Then, we compare the function $u_{n,\tau }^{g*}$ with $u_{\tau }^*$.

### Removing the noise

We start by stating the main result of the section.

#### Proposition 2.1

Let Assumptions (A1)–A4 hold and $s\in \mathbb {N}$. Let $\mathcal {E}_{n,\tau }^{(\textbf{y}_n)}$ be defined by (9), where $R^{(s)}_n$, $\Delta _n$ are defined by (10), (8) respectively, and let $u_{n,\tau }^*$, $u_{n,\tau }^{g*}$ be the minimizers of $\mathcal {E}_{n,\tau }^{(\textbf{y}_n)}$, $\mathcal {E}_{n,\tau }^{(\textbf{g}_n)}$ respectively. Assume that $\xi _i$ are iid, mean zero, sub-Gaussian random variables. Then, for all $\alpha >1$, there exists $\varepsilon _0>0$ and $C>0$ such that for any $\varepsilon , n$ satisfying (16) and $\tau >0$ we have

$$\begin{aligned} \left\| u_{n,\tau }^* - u_{n,\tau }^{g*} \right\| _{\text {L}^2(\mu _n)}&\le C \left( \sqrt{\frac{\log (n)}{n\varepsilon ^d}} + \frac{\varepsilon ^{2s}}{\tau } \right) \\ \left\| \Delta _n^{\frac{s}{2}} u_{n,\tau }^* - \Delta _n^{\frac{s}{2}} u_{n,\tau }^{g*} \right\| _{\text {L}^2(\mu _n)}&\le C\left( \sqrt{\frac{\log (n)}{n\varepsilon ^d\tau }} + \frac{\varepsilon ^{s}}{\tau }\right) \end{aligned}$$

with probability at least $1-C n^{-\alpha }$.

The proof of the proposition will be given at the end of the section. The strategy is to compare the Euler–Lagrange equations associated with minimising $\mathcal {E}_{n,\tau }^{(\textbf{y}_n)}$ and $\mathcal {E}_{n,\tau }^{(\textbf{g}_n)}$. In particular, we have

18 $$\begin{aligned} \frac{1}{2} \nabla _{\text {L}^2(\mu _n)} \mathcal {E}_{n,\tau }^{(\textbf{y}_n)}(u_n) = \tau \Delta _n^s u_n + u_n - \textbf{y}_n \end{aligned}$$

and therefore

19 $$\begin{aligned} \tau \Delta _n^s u_{n,\tau }^* + u_{n,\tau }^* - \textbf{y}_n&= 0\nonumber \\ \tau \Delta _n^s u_{n,\tau }^{g*} + u_{n,\tau }^{g*} - \textbf{g}_n&= 0. \end{aligned}$$

We let $w_{n,\tau }^* = u_{n,\tau }^* - u_{n,\tau }^{g*}$ then it follows that

$$\begin{aligned} \tau \Delta _n^s w_{n,\tau }^* + w_{n,\tau }^* - (\textbf{y}_n-\textbf{g}_n) = 0 \end{aligned}$$

and $w_{n,\tau }^*$ minimizes $\mathcal {E}_n^{(\textbf{y}_n-\textbf{g}_n)} = \mathcal {E}_n^{(\varvec{\xi }_n)}$ where $\varvec{\xi }_n = (\xi _1,\ldots , \xi _n)$. We can write

20 $$\begin{aligned} w_{n,\tau }^* = \left( \tau \Delta _n^s + \textrm{Id}\right) ^{-1} \varvec{\xi }_n. \end{aligned}$$

To obtain an estimate on $\Vert w_{n,\tau }^*\Vert _{\text {L}^2(\mu _n)}$ we use an ansatz $\tilde{w}_n$ and show $\Vert w_{n,\tau }^* - \tilde{w}_n\Vert _{\text {L}^2(\mu _n)}\le C \sqrt{\frac{\log (n)}{n\varepsilon ^d}}$ and $\Vert \tilde{w}_n\Vert _{\text {L}^2(\mu _n)}\le \frac{C\varepsilon ^{2s}}{\tau }$ with high probability. Our choice of ansatz is to assume that the diagonal part of $\Delta _n$ dominates and therefore $\Delta _n\approx \frac{2}{n\varepsilon ^2} D_n$ which leads to the choice,

21 $$\begin{aligned} \tilde{w}_n = \left( \tau \left( \frac{2}{n\varepsilon ^2} D_n\right) ^s + \textrm{Id}\right) ^{-1} \varvec{\xi }_n. \end{aligned}$$

This choice of ansatz is appropriate because $\Delta _n$ is increasingly sparse (for instance, if $\eta (t)$ is the indicator function on [0, 1] then we have positive edge weights only when $|x_i-x_j|\le \varepsilon$, and therefore each node has on the order of $n\varepsilon ^d$ edges, hence the fraction of non-zero entries in *W* is $\frac{n\varepsilon ^d}{n} = \varepsilon ^d$) and therefore well approximated by a diagonal matrix, which with high probability will not amplify the vector $\xi$. We can equivalently write

22 $$\begin{aligned} \tilde{w}_n(x_i) = \frac{\xi _i}{\tau \left( \frac{2}{n\varepsilon ^2} \sum _{k=1}^n W_{i,k}\right) ^s + 1}. \end{aligned}$$

The following lemmas will be useful.

#### Lemma 2.2

Under Assumptions (A1)–A4 there exists $C,C_1,C_2,c>0$ such that, if $n\varepsilon ^d\ge 1$ then

$$\begin{aligned} C_1 \le \frac{1}{n} \sum _{j=1}^n W_{i,j} \le C_2 \qquad \text {and} \qquad \#\left\{ j:\, W_{i,j}>0\right\} \le Cn\varepsilon ^d \end{aligned}$$

for all $i=1,\ldots , n$ with probability at least $1-2ne^{-cn\varepsilon ^d}$.

#### Proof

Fix $i\in \mathbb {N}$, then $W_{i,j}$ are iid for $j\ne i$. If $M=\Vert \eta _\varepsilon \Vert _{\textrm{L}^{\infty }(\mathbb {R})}$ and $\sigma ^2 = \mathbb {E}\left( W_{i,j} - \mathbb {E}[W_{i,j}]\right) ^2$ (where the expectation $\mathbb {E}[W_{i,j}]$ is taken over $x_j$) then it is straightforward to show the bounds $\sigma ^2\le C\varepsilon ^{-d}$ and $M\le C\varepsilon ^{-d}$. By Bernstein’s inequality, for all $t>0$,

$$\begin{aligned} \mathbb {P}\left( \left| \sum _{j\ne i} \left( W_{i,j} - \mathbb {E}[W_{i,j}]\right) \right| >t \right) \le 2\exp \left( -\frac{t^2}{2n\sigma ^2+\frac{4Mt}{3}}\right) \le 2\exp \left( -\frac{ct^2\varepsilon ^d}{n + t} \right) . \end{aligned}$$

Choosing $t=\lambda n$ and restricting to $\lambda \le 1$ we have

$$\begin{aligned} \mathbb {P}\left( \left| \sum _{j\ne i} \left( W_{i,j} - \mathbb {E}[W_{i,j}]\right) \right| >\lambda n \right) \le 2\exp \left( -cn\varepsilon ^d\lambda ^2 \right) . \end{aligned}$$

Hence, (recalling $$W_{i,i}=0$$)

$$\begin{aligned} (n-1) \mathbb {E}[W_{i,j}] - \lambda n \le \sum _{j=1}^n W_{i,j} \le (n-1) \mathbb {E}[W_{i,j}] + \lambda n \end{aligned}$$

with probability at least $1-2e^{-cn\varepsilon ^d\lambda ^2 }$. One can show that there exists $C_1,C_2$ such that $C_1\le \mathbb {E}[W_{i,j}] \le C_2$. For $n\ge 2$ (so that $n-1\ge n/2$),

$$\begin{aligned} \frac{C_1}{2} - \lambda \le \frac{1}{n} \sum _{j=1}^n W_{i,j} \le C_2 + \lambda \end{aligned}$$

with probability at least $1-2e^{-cn\varepsilon ^d\lambda ^2 }$. Choosing $\lambda = \frac{C_1}{4}$ and union bounding over $i\in \{1,\ldots ,n\}$ we can conclude the first result.

The second result follows from the first by choosing $\tilde{\varepsilon }=2\varepsilon$ and letting $\tilde{W}_{i,j} = \eta _{\tilde{\varepsilon }}(|x_i-x_j|)$. Then, $W_{i,j}>0$ implies $\tilde{\varepsilon }^d \tilde{W}_{ij}\ge 0.5$ and therefore $\#\{j{:}\,W_{i,j}>0\} \le 2\tilde{\varepsilon }^d \sum _{j=1}^n \tilde{W}_{i,j}$. Applying the first part of the lemma we have $2\tilde{\varepsilon }^d \sum _{j=1}^n \tilde{W}_{i,j}\le 2C_2n\tilde{\varepsilon }^d = 2^{d+1} C_2n\varepsilon ^d$ as required. $\square$

#### Lemma 2.3

Under Assumptions (A1)–A4 define $\Delta _n$ and $D_n$ by (8). Then, for all $\alpha >1$, there exists $\varepsilon _0>0$ and $C>0$ such that for all $\varepsilon , n$ satisfying (16) we have

$$\begin{aligned} \Vert \Delta _n\Vert _{\textrm{op}}\le \frac{C}{\varepsilon ^2}, \qquad \Vert D_n \Vert _{\textrm{op}} \le Cn, \qquad \text {and} \qquad \Vert W_n\Vert _{\textrm{op}} \le Cn \end{aligned}$$

with probability at least $1-Cn^{-\alpha }$.

#### Proof

For $d\ge 3$ one can bound $\Vert \Delta _n\Vert _{\textrm{op}}\le C\varepsilon ^{-2}$ by [18, Lemma 22]. Indeed, $\Vert \Delta _n\Vert _{\textrm{op}}\le C\varepsilon ^{-2}$ whenever $\textrm{d}_{\textrm{W}^\infty }(\mu _n,\mu )<\varepsilon$, where $\textrm{d}_{\textrm{W}^\infty }$ is the $\infty$-Wasserstein distance. By [77, Theorem 1.1] this holds with probability at least $1-Cn^{-\alpha }$. The same argument is used in (23) below as one step in the proof for $d=2$.

For $d=2$ a small modification is required to remove the additional logarithmic factors that are present in the scaling of $\textrm{d}_{\textrm{W}^\infty }(\mu _n,\mu )$, i.e. one has $\textrm{d}_{\textrm{W}^\infty }(\mu _n,\mu )\sim \frac{(\log (n))^{\frac{3}{4}}}{\sqrt{n}}$ and therefore requires $\varepsilon \ge C\frac{(\log (n))^{\frac{3}{4}}}{\sqrt{n}}$. However, this can be avoided by comparing $\mu _n$ to a smooth approximation of $\mu$.

In [78, Lemma 3.1] the authors show, in the Euclidean setting, that if $\varepsilon =\varepsilon _n\rightarrow 0$ satisfies $\frac{\log n}{n\varepsilon _n^d}\rightarrow 0$ then there exists an absolutely continuous probability measure $\tilde{\mu }_n\in \mathcal {P}(\Omega )$ such that

$$\begin{aligned} \frac{\textrm{d}_{\textrm{W}^\infty }(\mu _n,\tilde{\mu }_n)}{\varepsilon _n} \rightarrow 0 \qquad \text {and} \qquad \Vert \rho - \tilde{\rho }_n \Vert _{\text {L}^\infty (\mu )} \rightarrow 0 \end{aligned}$$

where $\tilde{\rho }_n$ is the density of $\tilde{\mu }_n$. As in [14, Proposition 2.10] the proof can be modified to give a non-asymptotic quantitative high probability bound. In particular, there exists constants *C*, $\varepsilon _0$ and $\theta _0$ such that if $n^{-\frac{1}{d}} \le \varepsilon \le \varepsilon _0$ and $\theta \le \theta _0$ then

$$\begin{aligned} \textrm{d}_{\textrm{W}^\infty }(\mu _n,\tilde{\mu }_n) \le \varepsilon \qquad \text {and} \qquad \Vert \rho - \tilde{\rho }_n \Vert _{\text {L}^\infty (\mu )} \le C(\theta +\varepsilon ) \end{aligned}$$

with probability at least $1-2ne^{-cn\theta ^2\varepsilon ^d}$. For the rest of the proof we fix $\theta =\theta _0$ and absorb it into the constants.

Note that if we define $\bar{\eta } = \eta ((|\cdot |-1)_+)$ and $T_n{:} \ \Omega \rightarrow \Omega$ satisfies $\Vert T_n - \textrm{Id}\Vert _{\text {L}^\infty (\Omega )}\le \varepsilon$ then

$$\begin{aligned} \eta \left( \frac{|x-T_n(y)|}{\varepsilon }\right)&\le \eta \left( \frac{\left( |x-y|-|T_n(y)-y|\right) _+}{\varepsilon }\right) \\&\le \eta \left( \left( \left| \frac{x-y}{\varepsilon }\right| -1\right) _+\right) \\&= \bar{\eta }\left( \frac{|x-y|}{\varepsilon }\right) . \end{aligned}$$

We choose $T_n$ to be a transport map satisfying $T_{n\#}\tilde{\mu }_n = \mu _n$ and $\Vert T_n-\textrm{Id}\Vert _{\text {L}^\infty (\tilde{\mu }_n)} = \textrm{d}_{\textrm{W}^\infty }(\mu _n,\tilde{\mu }_n)$ (since $\tilde{\mu }_n$ has a Lebesgue density then we may apply standard optimal transport results, in particular Brenier’s theorem, to infer existence of such a map).

Let $\lambda _{\max }$ be the largest eigenvalue of $\Delta _n$ then, as in the proof of [18, Lemma 22],

23 $$\begin{aligned} \begin{aligned} \lambda _{\max }&= \sup _{\Vert u\Vert _{\text {L}^2(\mu _n)}=1} \langle u,\Delta _n u\rangle _{\text {L}^2(\mu _n)} \\&\le \frac{4}{n^2\varepsilon ^{d+2}} \sup _{\Vert u\Vert _{\text {L}^2(\mu _n)}=1} \sum _{i,j=1}^n \eta \left( \frac{|x_i-x_j|}{\varepsilon }\right) u(x_i)^2 \\&= \frac{4}{n\varepsilon ^{d+2}} \sup _{\Vert u\Vert _{\text {L}^2(\mu _n)}=1} \sum _{i=1}^n u(x_i)^2 \int _\Omega \eta \left( \frac{|x_i-T_n(y)|}{\varepsilon }\right) \tilde{\rho }_n(y) \, \textrm{d}y \\&\le \frac{4}{n\varepsilon ^{d+2}} \sup _{\Vert u\Vert _{\text {L}^2(\mu _n)}=1} \sum _{i=1}^n u(x_i)^2 \int _\Omega \bar{\eta }\left( \frac{|x_i-y|}{\varepsilon }\right) \tilde{\rho }_n(y) \, \textrm{d}y \\&\le \frac{4}{n\varepsilon ^{d+2}} \sup _{\Vert u\Vert _{\text {L}^2(\mu _n)}=1} \sum _{i=1}^n u(x_i)^2 \int _\Omega \bar{\eta }\left( \frac{|x_i-y|}{\varepsilon }\right) \left( \rho (y) + C\right) \, \textrm{d}y \\&\le \frac{C}{\varepsilon ^2} \end{aligned} \end{aligned}$$

since $$\int _{\mathbb {R}^d} \bar{\eta }(|z|) \, \textrm{d}z <+\infty $$.

Although the bound holds for probability at least $1-Cne^{-cn\varepsilon ^d}$ when $d=2$ we can assume that the *C* in (16) is sufficiently large so that $\frac{n\varepsilon ^d}{\log (n)}\ge \frac{\alpha +1}{c}$. After some elementary algebra one has that $1-Cne^{-cn\varepsilon ^d}\ge 1-Cn^{-\alpha }$.

For any $v\in \text {L}^2(\mu _n)$ we have, by Lemma 2.2 with probability at least $1-2ne^{-cn\varepsilon ^d}$,

$$\begin{aligned} \Vert D_nv\Vert _{\text {L}^2(\mu _n)}^2 = \frac{1}{n} \sum _{i=1}^n \left( v(x_i) \sum _{j=1}^n W_{i,j}\right) ^2 \le C_2^2 n \sum _{i=1}^n v(x_i)^2 = C_2^2 n^2 \Vert v\Vert _{\text {L}^2(\mu _n)}^2 \end{aligned}$$

which implies $\Vert D_n\Vert _{\textrm{op}}\le C_2n$. The operator norm bound on $W_n$ follows from the bounds on the operator norms of $\Delta _n$, $D_n$ and the triangle inequality. Choosing *C* in Equation (16) sufficiently large we can assume that $1-2ne^{-cn\varepsilon ^d}\ge 1-Cn^{-\alpha }$. $\square$

In fact, [18, Lemma 22], suggests the operator bound on $\Delta _n$ is sharp (up to a constant), that is, there exists $c>0$ such that $\Vert \Delta _n \Vert _{\textrm{op}}\ge \frac{c}{\varepsilon ^2}$.

#### Lemma 2.4

Let Assumptions (A1)–A4 hold and $s\ge 1$, $k\in \mathbb {N}$. Let $\xi _i$ be iid, mean zero, sub-Gaussian random variables. Define $\tilde{w}_n$ by (22) and $D_n$ by (8). Then, for any $\alpha >1$ there exists $\varepsilon _0>0$ and $C>0$ such that for all $\varepsilon , n$ satisfying (16) and $\tau >0$ we have

$$\begin{aligned} \Vert W_n (D_n)^{k-1} \tilde{w}_n \Vert _{\text {L}^2(\mu _n)} \le \frac{Cn^k \varepsilon ^{2s}}{\tau } \sqrt{\frac{\log (n)}{n\varepsilon ^d}} \end{aligned}$$

with probability at least $1-Cn^{-\alpha }$.

#### Proof

Let us condition on a graph $G_n$ that satisfies the two inequalities:

(i) $$C_1\le \frac{1}{n} \sum _{j=1}^n W_{i,j} \le C_2$$, for all $$i=1,2,\ldots ,n$$,
(ii) $\#\left\{ j:\, W_{i,j}>0\right\} \le Cn\varepsilon ^d$ for all $i=1,2,\ldots ,n$.

Fix $i\in \{1,2,\ldots ,n\}$ and define

$$\begin{aligned} q_j = \frac{\tau W_{i,j}}{\varepsilon ^{2s} n^{k-1}}\left[ D_n^{k-1} \tilde{w}_n\right] _j. \end{aligned}$$

Conditioned on $G_n$ we have that $q_j$ are zero mean and independent random variables. Moreover, since

$$\begin{aligned} q_j = \frac{\tau W_{i,j} \left( \sum _{\ell =1}^n W_{j,\ell }\right) ^{k-1} \xi _j}{\varepsilon ^{2s} n^{k-1} \left( \tau \left( \frac{2}{n\varepsilon ^2} \sum _{\ell =1}^n W_{j,\ell }\right) ^s+1\right) } \end{aligned}$$

then we have $|q_j|\le \frac{C|\xi _j|}{\varepsilon ^d}$ so $q_j$ is sub-Gaussian and $\Vert q_j\Vert _{\psi _2}\le \frac{C\Vert \xi _j\Vert _{\psi _2}}{\varepsilon ^d}\lesssim \frac{1}{\varepsilon ^d}$ where $\Vert \cdot \Vert _{\psi _2}$ is the Birnbaum–Orlicz norm defined by

$$\begin{aligned} \Vert Q\Vert _{\psi _2}:= \inf \left\{ c\ge 0:\, \mathbb {E}e^{\frac{Q^2}{c^2}} \le 2 \right\} \quad \text {for a random variable } Q. \end{aligned}$$

By Hoeffding’s inequality, for any $t>0$

$$\begin{aligned} \mathbb {P}\left( \left| \sum _{j=1}^n q_j\right|>t | G_n\right)&\le \mathbb {P}\left( \left| \sum _{j\,:\, W_{i,j}>0} q_j \right|>t | G_n\right) \\&\le 2\exp \left( -\frac{ct^2}{\sum _{j\,:\,W_{i,j}>0} \Vert q_j\Vert _{\psi _2}^2}\right) \\&\le 2e^{-\frac{ct^2\varepsilon ^d}{n}}. \end{aligned}$$

We choose $t = \lambda \sqrt{\frac{n\log (n)}{\varepsilon ^d}}$ so

$$\begin{aligned} \frac{\tau }{\varepsilon ^{2s}n^{k-1}} \left| \left[ W_nD_n^{k-1}\tilde{w}_n\right] _i \right| = \left| \sum _{j=1}^n q_j\right| \le \lambda \sqrt{\frac{n\log (n)}{\varepsilon ^d}} \end{aligned}$$

with probability at least $1-2n^{-c\lambda ^2}$, conditioned on $G_n$. Union bounding and selecting $\lambda = \sqrt{\frac{\alpha +1}{c}}$, we then get that the above bound holds for all $i\in \{1,2,\ldots ,n\}$ with probability at least $n^{1-c\lambda ^2} = n^{-\alpha }$. Hence, after absorbing $\alpha$ into the constant *C*,

$$\begin{aligned} \left\| W_nD_n^{k-1}\tilde{w}_n\right\| _{\text {L}^2(\mu _n)} \le \left\| W_nD_n^{k-1}\tilde{w}_n\right\| _{\text {L}^\infty (\mu _n)} \le \frac{Cn^k\varepsilon ^{2s}}{\tau } \sqrt{\frac{\log (n)}{n\varepsilon ^d}} \end{aligned}$$

conditioned on $G_n$ with probability at least $1-2n^{-\alpha }$. Since, by Lemma 2.2, the probability of $G_n$ satisfying conditions (i) and (ii) is at least $1-2e^{-cn\varepsilon ^d}$, and by choosing *C* sufficiently large we have that $2e^{-cn\varepsilon ^d}\le Cn^{-\alpha }$ we can conclude the lemma. $\square$

To control $\Vert \tilde{w}_n-w_{n,\tau }^*\Vert _{\text {L}^2(\mu _n)}$ we take advantage of the convexity of our objective functional $\mathcal {E}_{n,\tau }^{(\varvec{\xi }_n)}$, where we recall that $\varvec{\xi }_n = \textbf{y}_n - \textbf{g}_n$. In particular, one can easily show that $\mathcal {E}_{n,\tau }^{(\varvec{\xi }_n)}$ satisfies

$$\begin{aligned}&\left\langle \nabla _{\text {L}^2(\mu _n)} \mathcal {E}_{n,\tau }^{(\varvec{\xi }_n)}(v_n) - \nabla _{\text {L}^2(\mu _n)} \mathcal {E}_{n,\tau }^{(\varvec{\xi }_n)}(u_n),v_n-u_n\right\rangle _{\text {L}^2(\mu _n)}\\&\quad = 2\tau \left\| \Delta _n^{\frac{s}{2}} (v_n-u_n) \right\| _{\text {L}^2(\mu _n)}^2 + 2 \Vert u_n-v_n\Vert _{\text {L}^2(\mu _n)}^2 \end{aligned}$$

for any $u_n,v_n\in \text {L}^2(\mu _n)$. Hence,

$$\begin{aligned} \Vert u_n - v_n \Vert _{\text {L}^2(\mu _n)}&\le \frac{1}{2} \left\| \nabla _{\text {L}^2(\mu _n)} \mathcal {E}_n^{(\varvec{\xi }_n)}(v_n) - \nabla _{\text {L}^2(\mu _n)} \mathcal {E}_n^{(\varvec{\xi }_n)}(u_n)\right\| _{\text {L}^2(\mu _n)} \\ \left\| \Delta _n^{\frac{s}{2}}(u_n - v_n) \right\| _{\text {L}^2(\mu _n)}&\le \frac{1}{2\sqrt{\tau }} \left\| \nabla _{\text {L}^2(\mu _n)} \mathcal {E}_n^{(\varvec{\xi }_n)}(v_n) - \nabla _{\text {L}^2(\mu _n)} \mathcal {E}_n^{(\varvec{\xi }_n)}(u_n)\right\| _{\text {L}^2(\mu _n)}. \end{aligned}$$

Applying this bound to $u_n=w_{n,\tau }^*$ and $v_n=\tilde{w}_n$, and using the optimality of $w_{n,\tau }^*$, we have

24 $$\begin{aligned} \Vert w_{n,\tau }^* - \tilde{w}_n \Vert _{\text {L}^2(\mu _n)}&\le \frac{1}{2} \left\| \nabla _{\text {L}^2(\mu _n)} \mathcal {E}_n^{(\varvec{\xi }_n)}(\tilde{w}_n)\right\| _{\text {L}^2(\mu _n)} \end{aligned}$$

25 $$\begin{aligned} \left\| \Delta _n^{\frac{s}{2}}(w_{n,\tau }^* - \tilde{w}_n) \right\| _{\text {L}^2(\mu _n)}&\le \frac{1}{2\sqrt{\tau }} \left\| \nabla _{\text {L}^2(\mu _n)} \mathcal {E}_n^{(\varvec{\xi }_n)}(\tilde{w}_n)\right\| _{\text {L}^2(\mu _n)}. \end{aligned}$$

The next lemma will bound these gradients in order to prove $\textrm{L}^{2}$ convergence rates.

#### Lemma 2.5

Let Assumptions (A1)–A4 hold and $s\in \mathbb {N}$. Let $\xi _i$ be iid, mean zero, sub-Gaussian, random variables. Define $\tilde{w}_n$ by (22) and $w_{n,\tau }^*$ by (20) where $\Delta _n$ is given by (8). Then, for any $\alpha >1$, there exists $C>0$ such that if $\varepsilon , n$ satisfy (16) and $\tau >0$ we have

$$\begin{aligned} \Vert \tilde{w}_n - w_{n,\tau }^* \Vert _{\text {L}^2(\mu _n)}&\le C\sqrt{\frac{\log (n)}{n\varepsilon ^d}} \\ \left\| \Delta _n^{\frac{s}{2}} \tilde{w}_n - \Delta _n^{\frac{s}{2}}w_{n,\tau }^* \right\| _{\text {L}^2(\mu _n)}&\le C\sqrt{\frac{\log (n)}{n\varepsilon ^d\tau }} \end{aligned}$$

with probability at least $1-Cn^{-\alpha }$.

#### Proof

By the definition of $\tilde{w}_n$, namely Equation (21) and the Fréchet derivative of $\mathcal {E}_{n,\tau }^{(\varvec{\xi }_n)}$, see (18) we have

$$\begin{aligned} \frac{1}{2} \nabla _{\text {L}^2(\mu _n)} \mathcal {E}_{n,\tau }^{(\varvec{\xi }_n)}(\tilde{w}_n)&= \left( \tau \Delta _n^s +\textrm{Id}\right) \tilde{w}_n - \varvec{\xi }_n \\&= \frac{2^s\tau }{n^s\varepsilon ^{2s}} \left( (D_n-W_n)^s - D_n^s\right) \tilde{w}_n \\&= \frac{2^s\tau }{n^s\varepsilon ^{2s}} \left( \left( \sum _{\chi \in \{0,1\}^s}\prod _{i=1}^s D_n^{\chi _i} (-W_n)^{1-\chi _i}\right) -D_n^s \right) {\tilde{w}}_n. \end{aligned}$$

Using the bounds from Lemmas 2.3 and 2.4 and their associated probability estimates, along with the fact that we have cancelled the $D_n^s$ term, we then may bound

$$\begin{aligned} \frac{1}{2} \left\| \nabla _{\text {L}^2(\mu _n)} \mathcal {E}_{n,\tau }^{(\varvec{\xi }_n)}(\tilde{w}_n) \right\| _{\text {L}^2(\mu _n)} \le \frac{2^s\tau }{n^s\varepsilon ^{2s}} \sum _{\chi \in \{0,1\}^s} \frac{Cn^s \varepsilon ^{2s}}{\tau } \sqrt{\frac{\log (n)}{n \varepsilon ^d}} \le C \sqrt{\frac{\log (n)}{n \varepsilon ^d}}. \end{aligned}$$

This concludes the proof. $\square$

Our final lemma before proving Proposition 2.1 is to bound $\Vert \tilde{w}_n\Vert _{\text {L}^2(\mu _n)}^2$.

#### Lemma 2.6

Let Assumptions (A1)–A4 hold and $s\in \mathbb {N}$. Let $\xi _i$ be iid, mean zero, sub-Gaussian, random variables. Define $\tilde{w}_n$ by (22). Then, for any $\alpha >1$, there exists $C>0$ such that for all $\varepsilon , n$ satisfying $n\varepsilon ^d\ge 1$ and $\tau >0$ we have

$$\begin{aligned} \Vert \tilde{w}_n \Vert _{\text {L}^2(\mu _n)} \le \frac{C\varepsilon ^{2s}}{\tau } \end{aligned}$$

with probability at least $1-n^{-\alpha }$.

#### Proof

By application of Lemma 2.2 we have

$$\begin{aligned} \Vert \tilde{w}_n \Vert _{\text {L}^2(\mu _n)}^2&= \frac{1}{n} \sum _{i=1}^n \frac{\xi _i^2}{\left( \tau \left( \frac{2}{n\varepsilon ^2} \sum _{k=1}^n W_{i,k}\right) ^s+1\right) ^2} \\&\le \frac{\varepsilon ^{4s}}{4^s C_1^{2s} \tau ^2 n} \sum _{i=1}^n \xi _i^2. \end{aligned}$$

Applying a Chernoff bound we have, for all $s,t\ge 0$,

$$\begin{aligned} \mathbb {P}\left( \sum _{i=1}^n \xi _i^2 \ge t \right) \le \frac{\mathbb {E}\left[ e^{s\sum _{i=1}^n \xi _i^2}\right] }{e^{st}} = \frac{\prod _{i=1}^n \mathbb {E}\left[ e^{s\xi _i^2}\right] }{e^{st}}. \end{aligned}$$

Choosing $s=\Vert \xi _i\Vert _{\Psi _2}^{-2}$ and $t=An$ we have

$$\begin{aligned} \mathbb {P}\left( \frac{1}{n} \sum _{i=1}^n \xi _i^2 \ge A\right) \le 2^n e^{-An\Vert \xi _i\Vert _{\Psi _2}^{-2}}. \end{aligned}$$

Now we choose *A* sufficiently large so that $\frac{A}{\Vert \xi _i\Vert _{\Psi _2}^2} \ge \alpha + \log 2$ and hence

$$\begin{aligned} 2^n e^{-An\Vert \xi _i\Vert _{\Psi _2}^{-2}} \le e^{-n\alpha } \le n^{-\alpha }. \end{aligned}$$

In particular, $\Vert \tilde{w}_n \Vert _{\text {L}^2(\mu _n)}\le \frac{C\varepsilon ^{2\,s}}{\tau }$ with probability at least $1-n^{-\alpha }$ as required. $\square$

The proof of Proposition 2.1 now follows from Lemmas 2.3, 2.5 and 2.6 since

$$\begin{aligned} \Vert u_{n,\tau }^* - u_{n,\tau }^{g*}\Vert _{\text {L}^2(\mu _n)} \le \Vert w_{n,\tau }^* - \tilde{w}_n \Vert _{\text {L}^2(\mu _n)} + \Vert \tilde{w}_n\Vert _{\text {L}^2(\mu _n)} \le C\left( \sqrt{\frac{\log (n)}{n\varepsilon ^d}} + \frac{\varepsilon ^{2s}}{\tau }\right) \end{aligned}$$

and

$$\begin{aligned} \left\| \Delta _n^{\frac{s}{2}} u_{n,\tau }^* - \Delta _n^{\frac{s}{2}} u_{n,\tau }^{g*}\right\| _{\text {L}^2(\mu _n)}&\le \left\| \Delta _n^{\frac{s}{2}} w_{n,\tau }^* - \Delta _n^{\frac{s}{2}} \tilde{w}_n \right\| _{\text {L}^2(\mu _n)} + \left\| \Delta _n^{\frac{s}{2}} \tilde{w}_n\right\| _{\text {L}^2(\mu _n)} \\&\le \left\| \Delta _n^{\frac{s}{2}} w_{n,\tau }^* - \Delta _n^{\frac{s}{2}} \tilde{w}_n \right\| _{\text {L}^2(\mu _n)} + \Vert \Delta _n\Vert _{\textrm{op}}^{\frac{s}{2}} \Vert \tilde{w}_n\Vert _{\text {L}^2(\mu _n)} \\&\le C\left( \sqrt{\frac{\log (n)}{n\varepsilon ^d\tau }} + \frac{\varepsilon ^{s}}{\tau }\right) \end{aligned}$$

with probability at least $1-Cn^{-\alpha }$.

### Discrete-to-continuum in the noiseless case

In this subsection we prove the following estimates which relate the functions $u_{n,\tau }^{g*}$ (the minimizer of $\mathcal {E}_{n,\tau }^{(\textbf{g}_n)}$ defined in (9) with $\textbf{a}_n=(g(x_1),\ldots ,g(x_n))$) with the function $u_{\tau }^*$ (the minimizer of $\mathcal {E}_{\infty ,\tau }^{(g)}$ defined in (14)).

As in (19) we can write the Euler–Lagrange equations associated with minimizing $\mathcal {E}_{\infty ,\tau }^{(g)}$ by

26 $$\begin{aligned} \tau \Delta _{\rho }^s u_\tau ^* + u_\tau ^* - g = 0. \end{aligned}$$

Our main result for this section is the following proposition.

#### Proposition 2.7

Let Assumptions (A1)–(A5) hold and $s\in \mathbb {N}$. Define $\Delta _n$, $\Delta _\rho$ and $\sigma _\eta$ by (8), (12) and (13) respectively. Let $u_{n,\tau }^{g*}$ and $u^*_\tau$ satisfy (19) and (26) respectively. Then, for any $\alpha >1$ and $\tau _0>0$ there exists constants $\varepsilon _0>0$, $C>c>0$ such that, for any $\varepsilon , n$ satisfying (16) and $\tau \in (0,\tau _0)$ we have

$$\begin{aligned} \left\| u_{n,\tau }^{g*} - u_{\tau }^{*}\lfloor _{\Omega _n}\right\| _{\textrm{L}^{2}(\mu _n)} \le C\tau \varepsilon \qquad \left\| \Delta _n^{\frac{s}{2}} u_{n,\tau }^{g*} - \Delta _\rho ^{\frac{s}{2}} u_{\tau }^{*}\lfloor _{\Omega _n}\right\| _{\textrm{L}^{2}(\mu _n)} \le C\varepsilon \end{aligned}$$

with probability at least $1-Cn^{-\alpha }-Cne^{-cn\varepsilon ^{d+4s}}$.

The proof of the proposition is given in Sect. 2.2.3. The proof of Theorem 1.1 follows immediately from the triangle inequality and Propositions 2.1 and 2.7. One of the main ingredients for proving Proposition 2.7 is the following result which is of interest on its own.

#### Theorem 2.8

Let Assumptions Assumptions (A1)–A4 hold and $s\in \mathbb {N}$. Define $\Delta _n$, $\Delta _\rho$ and $\sigma _\eta$ by (8), (12) and (13) respectively. Then, for any $\alpha >1$, there exists $C>c>0$ and $\varepsilon _0>0$ such that for any $u\in \textrm{C}^{2\,s+1}$ and $\varepsilon , n$ satisfying (16),

$$\begin{aligned} \left\| \left( \Delta _n^s - \Delta _\rho ^s \right) u \right\| _{\textrm{L}^{2}(\mu _n)} \le C \varepsilon \left( \Vert u\Vert _{\textrm{C}^{2s+1}(\Omega )} + 1 \right) \end{aligned}$$

with probability at least $1-Cn^{-\alpha } - Cne^{-cn\varepsilon ^{d+4s}}$.

We notice that when $s=1$ it is well known that the graph Laplacian is pointwise consistent and the rate at which it converges, e.g. [12]. Theorem 2.8 generalizes this result, and states that with high probability $\Delta _n^s u \rightarrow \Delta _\rho ^s u$ in an $\textrm{L}^{2}$ sense for all $s\in \mathbb {N}$ where *u* is sufficiently smooth and $\varepsilon = \varepsilon _n$ satisfies a lower bound. The proof of Theorem 2.8 is given in Sect. 2.2.2.

Before presenting a rigorous proof of Proposition 2.7, let us present a heuristic argument. First, we write

$$\begin{aligned} \Delta _n^s u(x) - \Delta _\rho ^s u(x)&= \Delta _n^{s-1} \left( \Delta _n - \Delta _\rho \right) v^{(0)}(x) + \left( \Delta _n^{s-1}-\Delta _\rho ^{s-1}\right) v^{(1)}(x) \\&= \Delta _n^{s-1} \left( \Delta _n - \Delta _\rho \right) v^{(0)}(x) + \Delta _n^{s-2} \left( \Delta _n - \Delta _\rho \right) v^{(1)}(x) \\&\qquad + \left( \Delta _n^{s-2}-\Delta _\rho ^{s-2}\right) v^{(2)}(x) \\&= \cdots \\&= \sum _{k=1}^s \Delta _n^{s-k} \left( \Delta _n - \Delta _\rho \right) v^{(k-1)}(x) \end{aligned}$$

where $v^{(k)} = \Delta _\rho ^k u$. We keep track of higher order errors in the pointwise consistency of the graph Laplacian, following the method in [15] to estimate, when $v\in \textrm{C}^{r}$,

27 $$\begin{aligned} \left( \Delta _n - \Delta _\rho \right) v(x) = \varepsilon E_1(x) + \varepsilon ^2 E_2(x) + \cdots \varepsilon ^{r-3} E_{r-3}(x) + \varepsilon ^{r-2} E_{r-2}(x) \end{aligned}$$

where $E_i\in \textrm{C}^{r-i-2}$. Now, heuristically one expects (with high probability) $\Vert \Delta _n^{j} E_i\Vert _{\textrm{L}^{2}(\mu _n)}\lesssim \Vert E_i\Vert _{\textrm{C}^{2j}(\Omega )}$ (when $j\le \frac{r-i-2}{2}$) and we recall a worse case (high probability) operator norm bound $\Vert \Delta _n^j \Vert _{\textrm{op}} \le C\varepsilon ^{-2j}$, see Lemma 2.3. Letting $u=u_\tau ^*$, and assuming $g\in \textrm{C}^{1}(\Omega )$, we can immediately infer that $u\in \textrm{C}^{2s+1}(\Omega )$ from (26) (as a standard elliptic regularity result). We choose $v=v^{(k-1)}$ in (27) and note that $r=2(s-k)+3$. Now, (with high probability)

$$\begin{aligned} \left\| \Delta _n^{s-k} E_i \right\| _{\textrm{L}^{2}(\Omega )}&= \left\| \Delta _n^{\frac{i-1}{2}} \Delta _n^{s-k-\frac{i-1}{2}} E_i \right\| _{\textrm{L}^{2}(\Omega )} \\&\le \left\| \Delta _n\right\| _{\textrm{op}}^{\frac{i-1}{2}} \left\| \Delta _n^{s-k-\frac{i-1}{2}} E_i\right\| _{\textrm{L}^{2}(\Omega )} \\&\le C\varepsilon ^{1-i} \left\| E_i\right\| _{\textrm{C}^{2(s-k)-i+1}(\Omega )}. \end{aligned}$$

So, (with high probability)

$$\begin{aligned} \left\| \Delta _n^{s-k} \left( \Delta _n - \Delta _\rho \right) v^{(k-1)} \right\| _{\textrm{L}^{2}(\mu _n)}&\le \sum _{i=1}^{2(s-k)+1} \varepsilon ^i \left\| \Delta _n^{s-k} E_i\right\| _{\textrm{L}^{2}(\mu _n)} \\&\le C\varepsilon \sum _{i=1}^{2(s-k)+1} \Vert E_i\Vert _{\textrm{C}^{2(s-k)-i+1}(\Omega )} \\&\le C\varepsilon . \end{aligned}$$

Thus, $\left\| \Delta _n^s u - \Delta _\rho ^s u \right\| _{\textrm{L}^{2}(\mu _n)} = O(\varepsilon )$ (note that *C* in the above inequality depends on *u*, in the proof we will show that this dependence is in terms of $\Vert u\Vert _{\textrm{C}^{2s+1}(\Omega )}$, i.e. $\left\| \Delta _n^s u - \Delta _\rho ^s u \right\| _{\textrm{L}^{2}(\mu _n)}\le C\varepsilon \left( \Vert u\Vert _{\textrm{C}^{2\,s+1}(\Omega )}+1\right)$).

The above discussion is clearly formal and we spend the remainder of the section making the proof rigorous. We do this in two stages. The first step gives operator bounds on $\Delta _n$ for smooth functions, i.e. quantifying $\Vert \Delta _n^{j} E_i\Vert _{\textrm{L}^{\infty }(\mu _n)}\lesssim \Vert E_i\Vert _{\textrm{C}^{2j}(\Omega )}$. The second step derives (27) from which we can prove Theorem 2.8 when combined with the first step.

#### Operator bounds on powers of the graph Laplacian

The aim of this subsection is to prove the following proposition.

##### Proposition 2.9

Let Assumptions (A1)–A4 hold and $m\in \mathbb {N}$. Define $\Delta _n$ by (8). Then, for all $\alpha >1$, there exists $C>c>0$ and $\varepsilon _0>0$ such that for any $\varepsilon , n$ satisfying (16) and for all $v\in \textrm{C}^{2m}(\Omega )$ we have

$$\begin{aligned} \Vert \Delta _n^m v\Vert _{\textrm{L}^{2}(\mu _n)} \le C (\Vert v\Vert _{\textrm{C}^{2m}(\Omega )}+1) \end{aligned}$$

with probability at least $1-Cne^{-cn\varepsilon ^{d+4m-2}}-Cn^{-\alpha }$.

Let us define the *non-local continuum Laplacian* by

28 $$\begin{aligned} \Delta _\varepsilon v(x) = \frac{2}{\varepsilon ^2} \int _{\Omega } \eta _{\varepsilon }(|x-y|) \left( v(x) - v(y) \right) \rho (y) \, \textrm{d}y. \end{aligned}$$

We prove the proposition in two steps. In the first step we show $\Vert \Delta _\varepsilon ^m v\Vert _{\textrm{L}^{2}(\Omega )}\le C \Vert v\Vert _{\textrm{C}^{2\,m}(\Omega )}$. In the second step we bound the difference $\Vert \Delta _n^m v - \Delta _\varepsilon ^m v\Vert _{\textrm{L}^{2}(\mu _n)}$. Initially we consider the case when $m=1$, which is just the difference of $\Delta _n v(x)$ to its expected value $\Delta _\varepsilon v(x) = \mathbb {E}\left[ \Delta _n v(x)\right]$. We can then bootstrap this to $m>1$. Putting the two steps together proves Proposition 2.9.

##### Lemma 2.10

Let Assumptions (A1), (A3) and A4 hold, and $k\in \mathbb {N}$. Define $\Delta _\varepsilon$ by (28). Then, there exists $C>0,\varepsilon _0>0$ such that for all $\varepsilon \in (0,\varepsilon _0)$ and for all $v\in \textrm{C}^{k+2}(\Omega )$ we have

29 $$\begin{aligned} \left\| \Delta _\varepsilon v \right\| _{\textrm{C}^{k}(\Omega )} \le C\Vert v\Vert _{\textrm{C}^{k+2}(\Omega )}. \end{aligned}$$

Furthermore, if $v\in \textrm{C}^{2k}(\Omega )$ then

30 $$\begin{aligned} \left\| \Delta _\varepsilon ^k v \right\| _{\textrm{C}^{0}(\Omega )} \le C\Vert v\Vert _{\textrm{C}^{2k}(\Omega )}. \end{aligned}$$

##### Proof

We can write, for $\varepsilon$ sufficiently small, where $\nabla$ above is the gradient in $\mathbb {R}^d$, and $D^2$ the matrix of second derivatives of a function on $\mathbb {R}^d$,

$$\begin{aligned} \Delta _\varepsilon v (x)&= \frac{2}{\varepsilon ^2} \int _{B(x,\varepsilon )} \eta _\varepsilon (|x-y|) (v(x) - v(y)) \rho (y) \,\textrm{d}y \\&= -\frac{2}{\varepsilon ^2} \int _{\mathbb {R}^d} \eta (|z|) \left( \varepsilon \nabla v(x) \cdot z + \varepsilon ^2 \int _0^1 \int _0^t D^2 v(x+\varepsilon sz)[z,z]\,\textrm{d}s \,\textrm{d}t \right) \\&\qquad \times \left( \rho (x) + \varepsilon \int _0^1 \nabla \rho (x+\varepsilon sz) \cdot z \,\textrm{d}s\right) \,\textrm{d}z, \end{aligned}$$

by Taylor’s theorem and a change of variables. Using the reflective symmetry of $\eta$ we have $\int _{\mathbb {R}^d} \eta (|z|) z \, \textrm{d}z=0$ and hence,

$$\begin{aligned} \Delta _\varepsilon v (x)&= -2\nabla v(x) \cdot \int _{\mathbb {R}^d} \eta (|z|) z \int _0^1 \nabla \rho (x+\varepsilon s z) \cdot z \, \textrm{d}s \, \textrm{d}z \\&\quad - 2\rho (x) \int _{\mathbb {R}^d} \eta (|z|) \int _0^1 \int _0^t D^2v(x+\varepsilon s z)[z,z] \, \textrm{d}s \,\textrm{d}t \, \textrm{d}z \\&\quad - 2 \varepsilon \int _{\mathbb {R}^d} \eta (|z|) \left( \int _0^1 \int _0^t D^2v(x+\varepsilon s z)[z,z] \, \textrm{d}s \, \textrm{d}t \right) \left( \int _0^1 \nabla \rho (x+\varepsilon s z) \cdot z \, \textrm{d}s \right) \, \textrm{d}z. \end{aligned}$$

If $v\in \textrm{C}^{k+2}(\Omega )$ and $\rho \in \textrm{C}^{k+1}(\Omega )$ then $\Delta _\varepsilon v\in \textrm{C}^{k}(\Omega )$ and moreover

$$\begin{aligned} \Vert \Delta _\varepsilon v\Vert _{\textrm{C}^{k}(\Omega )} \hspace{-2pt}&\le \hspace{-2pt} C \left( \Vert v\Vert _{\textrm{C}^{k+1}(\Omega )} \Vert \rho \Vert _{\textrm{C}^{k+1}(\Omega )} \hspace{-2.3pt} + \Vert v\Vert _{\textrm{C}^{k+2}(\Omega )} \Vert \rho \Vert _{\textrm{C}^{k}(\Omega )} \hspace{-2.3pt} + \varepsilon \Vert v\Vert _{\textrm{C}^{k+2}(\Omega )} \Vert \rho \Vert _{\textrm{C}^{k+1}(\Omega )} \right) \\&\le \hspace{-2pt} C \Vert v\Vert _{\textrm{C}^{k+2}(\Omega )}. \end{aligned}$$

This proves the first part of the lemma. Iterating the estimate (29) implies (30). $\square$

Now we turn to Step 2 and bounding the difference $\Delta _n-\Delta _\varepsilon$.

##### Lemma 2.11

Let Assumptions (A1)–A4 hold. Define $\Delta _n$ by (8) and $\Delta _\varepsilon$ by (28). For any $\varepsilon _0>0$ there exists $C>c>0$ such that for any $\varepsilon \in (0,\varepsilon _0)$, $p>0$, $n\in \mathbb {N}$ and $w\in \textrm{C}^{1}(\Omega )$ we have

$$\begin{aligned} \sup _{x\in \Omega _n} \left| \left( \Delta _n - \Delta _\varepsilon \right) w(x) \right| \le \varepsilon ^p \Vert w\Vert _{\textrm{C}^{1}(\Omega )} \end{aligned}$$

with probability at least $1-Cne^{-cn\varepsilon ^{d+2p+2}}$.

##### Proof

Fix $w\in \textrm{C}^{1}(\Omega )$, $x\in \Omega _n$ and let $\Xi _i = \frac{2}{\varepsilon ^2} \eta _\varepsilon (|x-y|) \left( w(x)-w(y)\right)$. So,

31 $$\begin{aligned} \frac{1}{n}\sum _{i=1}^n \Xi _i = \Delta _n w(x) \qquad \text {and} \qquad \mathbb {E}[\Xi _i] = \Delta _\varepsilon w(x). \end{aligned}$$

Note that

$$\begin{aligned} \left| \Xi _i-\mathbb {E}[\Xi _i]\right| \le \frac{C\Vert w\Vert _{\textrm{C}^{1}(\Omega )}}{\varepsilon ^{d+1}} \qquad \text {and} \qquad \mathbb {E}\left[ \Xi _i - \mathbb {E}[\Xi _i]\right] ^2 \le \frac{C\Vert w\Vert _{\textrm{C}^{1}(\Omega )}^2}{\varepsilon ^{d+2}}. \end{aligned}$$

By Bernstein’s inequality for any $t>0$,

$$\begin{aligned} \mathbb {P}\left( \sum _{i=1}^n \left( \Xi _i - \mathbb {E}[\Xi _i] \right) \ge t \right) \le \exp \left( -\frac{ct^2\varepsilon ^{d+2}}{n\Vert w\Vert _{\textrm{C}^{1}(\Omega )}^2 + t\varepsilon \Vert w\Vert _{\textrm{C}^{1}(\Omega )}}\right) . \end{aligned}$$

Choosing $$t = n\varepsilon ^{p}\Vert w\Vert _{\textrm{C}^{1}(\Omega )}$$ implies

$$\begin{aligned} \mathbb {P}\left( \sum _{i=1}^n \left( \Xi _i - \mathbb {E}[\Xi _i] \right) \ge n\varepsilon ^{p}\Vert w\Vert _{\textrm{C}^{1}(\Omega )} \right) \le \exp \left( -\frac{cn\varepsilon ^{d+2p+2}}{1 + \varepsilon ^{p+1}}\right) \le \exp \left( -cn\varepsilon ^{d+2p+2}\right) . \end{aligned}$$

Symmetrising the argument we have

$$\begin{aligned} \left| \sum _{i=1}^n \left( \Xi _i - \mathbb {E}[\Xi _i] \right) \right| \le n\varepsilon ^{p}\Vert w\Vert _{\textrm{C}^{1}(\Omega )} \end{aligned}$$

with probability at least $1-2e^{-cn\varepsilon ^{d+2p+2}}$. Substituting in (31) and union bounding over all $x\in \Omega _n$ we have proved the lemma. $\square$

Using the above lemma we can provide a bound on $\Delta _n^m-\Delta _\varepsilon ^m$.

##### Lemma 2.12

Assume Assumptions (A1)–A4 hold and $m\in \mathbb {N}$. Define $\Delta _n$ by (8) and $\Delta _\varepsilon$ by (28). Then, for all $\alpha >1$, there exists $C>c>0$ and $\varepsilon _0>0$ such that for any $\varepsilon , n$ satisfying (16) and $v\in \textrm{C}^{2m-1}(\Omega )$ we have

$$\begin{aligned} \left\| \Delta _n^m v - \Delta _\varepsilon ^m v \right\| _{\textrm{L}^{2}(\mu _n)} \le C\Vert v\Vert _{\textrm{C}^{2m-1}(\Omega )} \end{aligned}$$

with probability at least $1-Cne^{-cn\varepsilon ^{d+4m-2}}-Cn^{-\alpha }$.

##### Proof

We can write

$$\begin{aligned} \left\| \Delta _n^m v - \Delta _\varepsilon ^m v \right\| _{\textrm{L}^{2}(\mu _n)}&\le \sum _{i=0}^{m-1} \left\| \Delta _n^{m-i} \Delta _\varepsilon ^i v - \Delta _n^{m-i-1} \Delta _\varepsilon ^{i+1} v \right\| _{\textrm{L}^{2}(\mu _n)} \\&\le \sum _{i=0}^{m-1} \Vert \Delta _n \Vert _{\textrm{op}}^{m-i-1} \left\| \left( \Delta _n - \Delta _\varepsilon \right) \Delta _\varepsilon ^i v \right\| _{\textrm{L}^{2}(\mu _n)} \\&\le C\sum _{i=0}^{m-1} \Vert \Delta _\varepsilon ^i v\Vert _{\textrm{C}^{1}(\Omega )} \\&\le C\sum _{i=0}^{m-1} \Vert v\Vert _{\textrm{C}^{2i+2}(\Omega )} \\&\le C\Vert v\Vert _{\textrm{C}^{2m}(\Omega )} \end{aligned}$$

by Lemmas 2.3,  2.10 and 2.11 with probability at least $1-Cne^{-cn\varepsilon ^{d+4m-2}}-Cn^{-\alpha }$. $\square$

#### Proof of Theorem 2.8

Now, we note that

$$\begin{aligned} \Delta _n^s u(x) - \Delta _\rho ^s u(x)&= \Delta _n^{s-1} \left( \Delta _n - \Delta _\rho \right) v^{(0)}(x) + \left( \Delta _n^{s-1}-\Delta _\rho ^{s-1}\right) v^{(1)}(x) \\&= \Delta _n^{s-1} \left( \Delta _n - \Delta _\rho \right) v^{(0)}(x) + \Delta _n^{s-2} \left( \Delta _n - \Delta _\rho \right) v^{(1)}(x) \\&\qquad + \left( \Delta _n^{s-2}-\Delta _\rho ^{s-2}\right) v^{(2)}(x) \\&= \cdots \\&= \sum _{k=1}^s \Delta _n^{s-k} \left( \Delta _n - \Delta _\rho \right) v^{(k-1)}(x) \end{aligned}$$

where $$v^{(i)} = \Delta _\rho ^i u$$.

The idea is now to use pointwise convergence but to keep track of higher order terms than the estimates that appear in [12, 15]. For example, [15] shows that if $f\in \textrm{C}^{3}$ then

32 $$\begin{aligned} \left| \Delta _n f(x) - \Delta _{\rho } f(x) \right| \le C\Vert f\Vert _{\textrm{C}^{3}} \vartheta , \end{aligned}$$

where $\vartheta \ge \varepsilon$, with probability at least $1-Cne^{-cn\varepsilon ^{d+2}\vartheta ^2}$. Directly applying the operator bounds we have

$$\begin{aligned} \left\| \Delta _n^s u - \Delta _\rho ^s u \right\| _{\textrm{L}^{2}(\mu _n)}&\le \sum _{k=1}^s \Vert \Delta _n \Vert _{\textrm{op}}^{s-k} \left\| \left( \Delta _n - \Delta _\rho \right) v^{(k-1)} \right\| _{\textrm{L}^{2}(\Omega )} \\&\le C\sum _{k=1}^s \varepsilon ^{-2(s-k)} \Vert v^{(k-1)}\Vert _{\textrm{C}^{3}(\Omega )} \vartheta _k \\&\le C\Vert u\Vert _{\textrm{C}^{2s+1}} \sum _{k=1}^s \varepsilon ^{-2(s-k)} \vartheta _k. \end{aligned}$$

If we could choose $\vartheta _k = \varepsilon ^{1+2(s-k)}$ then the proof is immediate; however the pointwise convergence result (32) requires $\vartheta \ge \varepsilon$ which rules out this choice. However, we will show that this gives the right answer, in particular, that the convergence is within $\varepsilon$ with probability at least $1-Cne^{-cn\varepsilon ^{d+4s}}$. The rest of the section is devoted to removing the assumption that $\vartheta _k\ge \varepsilon$.

##### Proof of Theorem 2.8

Let us fix *k* and write $v = v^{(k-1)}$. Then, assuming $u\in \textrm{C}^{2\,s+1}(\Omega )$ we have $v\in \textrm{C}^{2(s-k)+3}(\Omega )$ and so, for *y* sufficiently close to *x*,

$$\begin{aligned} v(y) = v(x) + \sum _{j=1}^{2(s-k+1)} \sum _{i^{(j)}\in \{1,\ldots ,d\}^j} a_{i^{(j)}}^{(j)} \prod _{\ell = 1}^j \left( y_{i_\ell ^{(j)}} - x_{i_\ell ^{(j)}} \right) + O\left( \left| y_{i_\ell ^{(j)}} - x_{i_\ell ^{(j)}}\right| ^{2(s-k)+3}\right) \end{aligned}$$

where

$$\begin{aligned} a_{i^{(j)}}^{(j)} = \frac{1}{j!} \frac{\partial ^j v}{\partial x_{i_1^{(j)}}\cdots \partial x_{i_j^{(j)}}}(x), \end{aligned}$$

$i^{(j)} = (i_1^{(j)},\ldots ,i_j^{(j)}) \in \{1,\ldots d\}^j$ and the big-*O* notation is understood as meaning that there exists a bounded function, say $\Xi _{i_\ell ^{(j)}}$ depending on $x_{i_\ell ^{(j)}}$ and $y_{i_\ell ^{(j)}}$ such that $O\left( \left| y_{i_\ell ^{(j)}} - x_{i_\ell ^{(j)}}\right| ^{2(s-k)+3}\right) = \Xi _{i_\ell ^{(j)}}(x_{i_\ell ^{(j)}},y_{i_\ell ^{(j)}})|y_{i_\ell ^{(j)}}-x_{i_\ell ^{(j)}}|^{2(s-k)+3}$. Now we can write

$$\begin{aligned} \Delta _n v(x)&= \frac{2}{n\varepsilon ^2} \sum _{y\in \Omega _n} W_{xy} (v(x) - v(y)) \\&= - \frac{2}{n\varepsilon ^2} \sum _{y\in \Omega _n} W_{xy} \left[ \sum _{j=1}^{2(s-k+1)} \sum _{i^{(j)} \in \{1,\ldots d\}^j} a_{i^{(j)}}^{(j)} \prod _{\ell =1}^j \left( y_{i_\ell ^{(j)}} - x_{i_\ell ^{(j)}}\right) \right] \\&\quad + O\left( \frac{\varepsilon ^{2(s-k)+1}}{n} \sum _{y\in \Omega _n} W_{xy}\right) . \end{aligned}$$

By Lemma 2.2, $\frac{1}{n} \sum _y W_{xy} \le C$ for all $x\in \Omega _n$ with probability at least $1-2ne^{-cn\varepsilon ^d}$, hence we can write (with probability at least $1-2ne^{-cn\varepsilon ^d}$)

$$\begin{aligned} \Delta _n v(x) = -\sum _{j=1}^{2(s-k+1)} \sum _{i^{(j)} \in \{1,\ldots d\}^j} a_{i^{(j)}}^{(j)} I_{i^{(j)}}^{(j)} + O(\varepsilon ^{2(s-k)+1}) \end{aligned}$$

where

$$\begin{aligned} I_{i^{(j)}}^{(j)} = \sum _{y\in \Omega _n} \Psi _{i^{(j)}}^{(j)}, \qquad \Psi _{i^{(j)}}^{(j)}(y) = \frac{2}{n\varepsilon ^2} W_{xy} \prod _{\ell =1}^j \left( y_{i_\ell ^{(j)}}^{(j)} - x_{i_\ell ^{(j)}}^{(j)} \right) . \end{aligned}$$

Note that $\Vert \Psi _{i^{(j)}}^{(j)}\Vert _{\textrm{L}^{\infty }}\le \frac{C\varepsilon ^{j-2-d}}{n}$ and $\mathbb {E}[\Psi _{i^{(j)}}^{(j)}(Y)^2]\le \frac{C\varepsilon ^{2(j-2)-d}}{n^2}$, which follows from

$$\begin{aligned} |\Psi _{i^{(j)}}^{(j)}(y)|&\lesssim \frac{1}{n\varepsilon ^2} \underbrace{W_{xy}}_{\lesssim \frac{1}{\varepsilon ^d}} \prod _{\ell =1}^j \left( \underbrace{y^{(j)}_{i_\ell ^{(j)}} - x^{(j)}_{i_\ell ^{(j)}}}_{\lesssim \varepsilon } \right) \lesssim \frac{1}{n\varepsilon ^{2+d-j}} \\ \mathbb {E}[\Psi _{i^{(j)}}^{(j)}(Y)^2]&\lesssim \frac{1}{n^2\varepsilon ^4} \mathbb {E}\left[ \underbrace{W_{xy}^2}_{\lesssim \frac{1}{\varepsilon ^{2d}}} \prod _{\ell =1}^j \underbrace{\left( y_{i_\ell ^{(j)}}^{(j)} - x_{i_\ell ^{(j)}}^{(j)} \right) ^2}_{\varepsilon ^2} \right] \\&\lesssim \frac{1}{n^2\varepsilon ^{4+2d-2j}} \underbrace{\int \int _{|x-y|\le \varepsilon } \, \textrm{d}x \, \textrm{d}y}_{\lesssim \varepsilon ^d} \\&\lesssim \frac{1}{n^2\varepsilon ^{4+d-2j}}. \end{aligned}$$

Hence, by Bernstein’s inequality

$$\begin{aligned} I_{i^{(j)}}^{(j)}&= \frac{2}{\varepsilon ^2} \int _{\Omega } \eta _\varepsilon (|x-y)|) \left[ \prod _{\ell =1}^j \left( y_{i_\ell ^{(j)}} - x_{i_\ell ^{(j)}} \right) \right] \rho (y) \, \textrm{d}y + O(\varepsilon ^{j-2}\vartheta ) \\&= 2\varepsilon ^{j-2} \int _{\mathbb {R}^d} \eta (|z|) \left[ \prod _{\ell =1}^j z_{i_\ell ^{(j)}}\right] \rho (x+\varepsilon z) \, \textrm{d}z + O(\varepsilon ^{j-2}\vartheta ) \end{aligned}$$

with probability at least $1-2ne^{-cn\varepsilon ^d\vartheta ^2}$ for all $x\in \Omega _n$. After union bounding we may assume that the above estimate holds for all $x\in \Omega _n$, for all $k=1,\ldots ,s$, for all $j=1,\ldots , k$, and for all $i^{(j)}\in \{1,\ldots ,d\}^j$ with probability at least $1-Cne^{-cn\varepsilon ^d\vartheta ^2}$. We choose $\vartheta = \varepsilon ^{2(s-k)+3-j}$ and so, since $\vartheta \ge \varepsilon ^{2\,s}$, the following holds with probability at least $1-Cne^{-cn\varepsilon ^{d+4s}}$.

Now we approximate

$$\begin{aligned} \rho (x+\varepsilon z) = \sum _{m=0}^{2(s-k+1)-j} \varepsilon ^m \sum _{p^{(m)}\in \{1,\ldots ,d\}^m} b_{p^{(m)}}^{(m)} \prod _{q=1}^m z_{p_q^{(m)}} + O(\varepsilon ^{2(s-k)-j+3}) \end{aligned}$$

where

$$\begin{aligned} b_{p^{(m)}}^{(m)} = \frac{1}{m!} \frac{\partial ^m \rho }{\partial x_{p_1^{(m)}} \cdots \partial x_{p_m^{(m)}}}(x). \end{aligned}$$

Hence,

$$\begin{aligned} I_{i^{(j)}}^{(j)}&= 2 \sum _{m=0}^{2(s-k+1)-j} \sum _{p^{(m)}\in \{1,\ldots ,d\}^m} \varepsilon ^{m+j-2} b_{p^{(m)}}^{(m)} \int _{\mathbb {R}^d} \eta (|z|) \left[ \prod _{\ell =1}^j z_{i_\ell ^{(j)}}\right] \left[ \prod _{q=1}^m z_{p_q^{(m)}} \right] \, \textrm{d}z \\&\quad + O(\varepsilon ^{(2(s-k)+1}). \end{aligned}$$

Let

$$\begin{aligned} F(j,m) = \sum _{i^{(j)}\in \{1,\ldots ,d\}^j} \sum _{p^{(m)}\in \{1,\ldots ,d\}^m} a_{i^{(j)}}^{(j)} b_{p^{(m)}}^{(m)} C(i^{(j)},p^{(m)}) \end{aligned}$$

and

$$\begin{aligned} C(i^{(j)},p^{(m)}) = -2 \int _{\mathbb {R}^d} \eta (|z|) \left[ \prod _{\ell =1}^j z_{i_\ell ^{(j)}} \right] \left[ \prod _{q=1}^m z_{p_q^{(m)}} \right] \, \textrm{d}z \end{aligned}$$

so that

$$\begin{aligned} \Delta _n v(x) = \sum _{j=1}^{2(s-k+1)} \sum _{m=0}^{2(s-k+1)-j} \varepsilon ^{m+j-2} F(j,m) + O(\varepsilon ^{(2(s-k)+1}). \end{aligned}$$

We now look at the following terms: (i) $j=1$, $m=0$; (ii) $j=1$, $m=1$; and (iii) $j=2$, $m=0$ (the terms which are potentially of order $\varepsilon ^{-1}$ and $\varepsilon ^0$). For (i),

$$\begin{aligned} C(i,\emptyset ) = -2 \int _{\mathbb {R}^d} \eta (|z|) z_i \, \textrm{d}z = 0. \end{aligned}$$

For (ii),

$$\begin{aligned} C(i,p) = -2 \int _{\mathbb {R}^d} \eta (|z|) z_iz_p \, \textrm{d}x = \left\{ \begin{array}{ll} 0 &{} \text {if } i\ne p \\ -2\sigma _\eta &{} \text {if } i=p. \end{array} \right. \end{aligned}$$

For (iii),

$$\begin{aligned} C((i_1,i_2),\emptyset ) = -2 \int _{\mathbb {R}^d} \eta (|z|) z_{i_1} z_{i_2} \, \textrm{d}z = \left\{ \begin{array}{ll} 0 &{} \text {if } i_1\ne i_2 \\ -2 \sigma _\eta &{} \text {if } i_1=i_2. \end{array} \right. \end{aligned}$$

So $$F(1,0) = 0$$,

$$\begin{aligned} F(1,1) = -2 \sigma _\eta \sum _{i=1}^d a_i^{(1)} b_i^{(1)} = -2\sigma _\eta \nabla v(x) \cdot \nabla \rho (x), \end{aligned}$$

and

$$\begin{aligned} F(2,0) = -2 \sigma _\eta \sum _{i=1}^d a_{i,i}^{(2)} b^{(0)} = -\sigma _\eta \rho (x) \textrm{trace}(D^2 v(x)). \end{aligned}$$

As $F(1,0)\varepsilon ^{-1} + F(1,1) + F(2,0) = -\frac{\sigma _\eta }{\rho (x)} \textrm{div}(\rho ^2 \nabla v)(x) = \Delta _\rho v(x)$ then we have (adding back the *k* dependence on *v*)

$$\begin{aligned} \Delta _n v^{(k-1)}(x) - \Delta _\rho v^{(k-1)}(x)&= \sum _{m=2}^{2(s-k)+1} \varepsilon ^{m-1} F(1,m) + \sum _{m=1}^{2(s-k)} \varepsilon ^{m} F(2,m) \\&\quad + \sum _{j=3}^{2(s-k+1)} \sum _{m=0}^{2(s-k+1)-j} \varepsilon ^{m+j-2} F(j,m) + O(\varepsilon ^{2(s-k)+1}). \end{aligned}$$

In particular, if we let $F_{j,m}^{(k)}(x) = F(j,m)$ and define $E^{(k)}(x)$ to satisfy $O(\varepsilon ^{2(s-k)+1}) = \varepsilon ^{2(s-k)+1} E^{(k)}(x)$ then

$$\begin{aligned} \left\| \left( \Delta _n^s - \Delta _\rho ^s\right) u \right\| _{\textrm{L}^{2}(\mu _n)}&\le \sum _{k=1}^s \left\| \Delta _n^{s-k} \left( \Delta _n-\Delta _\rho \right) v^{(k-1)}\right\| _{\textrm{L}^{2}(\mu _n)} \\&\le \sum _{k=1}^s \sum _{m=2}^{2(s-k)+1} \varepsilon ^{m-1} \left\| \Delta _n^{s-k} F_{1,m}^{(k)} \right\| _{\textrm{L}^{2}(\mu _n)} \\&\quad + \sum _{k=1}^s \sum _{m=1}^{2(s-k)} \varepsilon ^{m} \left\| \Delta _n^{s-k} F_{2,m}^{(k)} \right\| _{\textrm{L}^{2}(\mu _n)} \\&\quad + \sum _{k=1}^s \sum _{j=3}^{2(s-k+1)} \sum _{m=0}^{2(s-k+1)-j} \varepsilon ^{m+j-2} \left\| \Delta _n^{s-k} F_{j,m}^{(k)} \right\| _{\textrm{L}^{2}(\mu _n)} \\&\quad + \sum _{k=1}^s \varepsilon ^{2(s-k)+1} \left\| \Delta _n^{s-k} E^{(k)} \right\| _{\textrm{L}^{2}(\mu _n)}. \end{aligned}$$

By Lemma 2.3 (with probability at least $1-Cn^{-\alpha }$) we have

$$\begin{aligned} \varepsilon ^{2(s-k)+1} \left\| \Delta _n^{s-k} E^{(k)} \right\| _{\textrm{L}^{2}(\mu _n)} \le \varepsilon ^{2(s-k)+1} \Vert \Delta _n \Vert _{\textrm{op}}^{s-k} \Vert E^{(k)} \Vert _{\textrm{L}^{2}(\mu _n)} \le \varepsilon \Vert E^{(k)} \Vert _{\textrm{L}^{2}(\mu _n)}. \end{aligned}$$

We also have $F_{j,m}^{(k)}\in \textrm{C}^{2(s-k)+3-j}$ and $\Vert F_{j,m}^{(k)}\Vert _{\textrm{C}^{2(s-k)+3-j}(\Omega )} \le C\Vert u\Vert _{\textrm{C}^{2\,s+1}(\Omega )}$, therefore we have for $j\ge 3$

$$\begin{aligned} \varepsilon ^{m+j-2} \left\| \Delta _n^{s-k} F_{j,m}^{(k)} \right\| _{\textrm{L}^{2}(\mu _n)}&\le \varepsilon ^{m+j-2} \Vert \Delta _n\Vert _{\textrm{op}}^{\frac{j-3}{2}} \left\| \Delta _n^{\frac{2(s-k)+3-j}{2}} F_{j,m}^{(k)} \right\| _{\textrm{L}^{2}(\mu _n)} \\&\le C \varepsilon ^{m+1} \left( \left\| F_{j,m}^{(k)}\right\| _{\textrm{C}^{2(s-k)+3-j}(\Omega )} + 1 \right) \end{aligned}$$

with probability at least $1-Cn^{-\alpha }-Cne^{-cn\varepsilon ^{d+4\,m-2}}\ge 1-Cn^{-\alpha }-Cne^{-cn\varepsilon ^{d+2\,s}}$ by Lemma 2.3 and Proposition 2.9. When $j=1,2$ we have, directly from Proposition 2.9,

$$\begin{aligned} \left\| \Delta _n^{s-k} F_{j,m}^{(k)} \right\| _{\textrm{L}^{2}(\mu _n)} \le \left\| F_{j,m}^{(k)} \right\| _{\textrm{C}^{2(s-k)}(\Omega )} \le \left\| F_{j,m}^{(k)} \right\| _{\textrm{C}^{2(s-k)+3-j}(\Omega )} \le C \Vert u\Vert _{\textrm{C}^{2s+1}(\Omega )} \end{aligned}$$

with probability at least $1-Cne^{-cn\varepsilon ^{d+2s}}$. Hence,

$$\begin{aligned} \left\| \left( \Delta _n^s - \Delta _\rho ^s\right) u \right\| _{\textrm{L}^{2}(\mu _n)} \le C\varepsilon \left( \Vert u\Vert _{\textrm{C}^{2s+1}(\Omega )} + 1 \right) \end{aligned}$$

with probability at least $1-Cn^{-\alpha } - Cne^{-cn\varepsilon ^{d+4s}}$. $\square$

##### Remark 11

In our proofs we avoid attempting to establish pointwise consistency results for the difference $\Delta _n^s - \Delta _\rho ^s$ (for arbitrary $s\in {\mathbb {N}}$) when acting on smooth enough functions, and instead by careful manipulation of the equations, we rely only on the existing pointwise consistency results for the case $s=1$ [11, 15].

#### Proof of Proposition 2.7

We start with two preliminary lemmas which will be used in the proof of Proposition 2.7.

##### Lemma 2.13

Let $\tau >0$, $s>0$, $\Delta _n$ be defined by (8) and $\Delta _\rho$ defined by (12) where $\sigma _\eta$ is defined by (13). Assume $w_n$ and *w* solve

$$\begin{aligned} \tau \Delta _n^s w_n + w_n&= h_n \\ \tau \Delta _\rho ^s w + w&= h \end{aligned}$$

for $h_n\in \textrm{L}^{2}(\mu _n)$ and $h\in \textrm{L}^{2}(\mu )$. Then,

$$\begin{aligned} \Vert w_n\Vert _{\textrm{L}^{2}(\mu _n)}&\le \Vert h_n\Vert _{\textrm{L}^{2}(\mu _n)} \\ \Vert w\Vert _{\textrm{L}^{2}(\mu )}&\le \Vert h\Vert _{\textrm{L}^{2}(\mu )}. \end{aligned}$$

##### Proof

Let $\{q_i^{(n)}\}_{i=1}^n$ be an eigenbasis of $\Delta _n$ with non-negative eigenvalues $\{\lambda _i^{(n)}\}_{i=1}^n$. Then $w_n$ solving $\tau \Delta _n^s w_n + w_n = h_n$ implies

$$\begin{aligned} \left( \tau [\lambda _i^{(n)}]^s + 1 \right) \langle w_n,q_i^{(n)}\rangle _{\textrm{L}^{2}(\mu _n)} = \langle h_n, q_i^{(n)}\rangle _{\textrm{L}^{2}(\mu _n)}. \end{aligned}$$

So,

$$\begin{aligned} \Vert w_n\Vert ^2_{\textrm{L}^{2}(\mu _n)}&= \sum _{i=1}^n \left| \left\langle w_n,q_i^{(n)}\right\rangle _{\textrm{L}^{2}(\mu _n)} \right| ^2 \\&= \sum _{i=1}^n \left| \frac{\left\langle h_n,q_i^{(n)}\right\rangle _{\textrm{L}^{2}(\mu _n)}}{1+\tau \left[ \lambda _i^{(n)}\right] ^s} \right| ^2 \\&\le \sum _{i=1}^n \left| \left\langle h_n,q_i^{(n)}\right\rangle _{\textrm{L}^{2}(\mu _n)} \right| ^2 \\&= \Vert h_n\Vert ^2_{\textrm{L}^{2}(\mu _n)}. \end{aligned}$$

The proof for $\Vert w\Vert _{\textrm{L}^{2}(\mu )} \le \Vert h\Vert _{\textrm{L}^{2}(\mu )}$ is analogous. $\square$

##### Lemma 2.14

Assume Assumptions (A3) and (A5) hold and $s>0$. Define $\Delta _\rho$ by (12) where $\sigma _\eta$ is defined by (13). Let $u_\tau ^*$ be the solution to (26). Then, for all $\tau _0>0$ there exists *C* such that

$$\begin{aligned} \sup _{\tau \in (0,\tau _0)} \Vert u_\tau ^{*}\Vert _{\textrm{C}^{2s+1}(\Omega )} \le C. \end{aligned}$$

##### Proof

Let $\{(\lambda _i,q_i)\}_{i=1}^\infty$ be eigenpairs of $\Delta _\rho$. Define $\mathcal {H}^k(\Omega ) = \left\{ u\in \textrm{L}^{2}(\mu ) {:}\, \sum _{i=1}^\infty \lambda _i^k \langle u,q_i\rangle _{\textrm{L}^{2}(\mu )}^2 < +\infty \right\}$ with the norm $\Vert u\Vert _{\mathcal {H}^k(\Omega )}^2 = \sum _{i=1}^\infty \lambda _i^k \langle u,q_i\rangle _{\textrm{L}^{2}(\mu )}^2$. And let $\textrm{H}^{k}(\Omega )$ be the usual Sobolev space with square integrable *k*th (weak) derivative. By [18, Lemma 17] $\mathcal {H}^k(\Omega )\subseteq \textrm{H}^{k}(\Omega )$ and there exists $C>c>0$ (depending only on the choice of *k*) such that

$$\begin{aligned} C\Vert u\Vert _{\mathcal {H}^k(\Omega )} \ge \Vert u\Vert _{\textrm{H}^{k}(\Omega )} \ge c\Vert u\Vert _{\mathcal {H}^k(\Omega )} \qquad \text {for all } u \in \mathcal {H}^k(\Omega ). \end{aligned}$$

As in the proof of Lemma 2.13 we take advanatge of the fact that $\langle u_\tau ^*,q_i\rangle _{\textrm{L}^{2}(\mu )} = \frac{\langle g,q_i\rangle _{\textrm{L}^{2}(\mu )}}{1+\tau \lambda _i^s}$ to infer

$$\begin{aligned} \Vert u_\tau ^*\Vert _{\mathcal {H}^k(\Omega )}^2&= \sum _{i=1}^\infty \lambda _i^k \langle u_\tau ^*,q_i\rangle _{\textrm{L}^{2}(\mu )}^2 \\&= \sum _{i=1}^\infty \lambda _i^k \left| \frac{\langle g,q_i\rangle _{\textrm{L}^{2}(\mu )}}{1+\tau \lambda _i^s} \right| ^2 \\&\le \sum _{i=1}^\infty \lambda _i^k \langle q,q_i\rangle _{\textrm{L}^{2}(\mu )}^2 \\&= \Vert g\Vert _{\mathcal {H}^k(\Omega )}^2 \\&\le C^2 \Vert g\Vert _{\textrm{H}^{k}(\Omega )}^2. \end{aligned}$$

Hence $\Vert u_\tau ^*\Vert _{\textrm{H}^{k}(\Omega )} \le \frac{C}{c} \Vert g\Vert _{\textrm{H}^{k}(\Omega )}$. By choosing *k* sufficiently large and employing Morrey’s inequality we can find $\bar{C}$ such that $\Vert u\Vert _{\textrm{C}^{2s+1}(\Omega )}\le \bar{C} \Vert u\Vert _{\textrm{H}^{k}(\Omega )}$ for all $u\in \textrm{H}^{k}(\Omega )$. In particular, $\Vert u_\tau ^*\Vert _{\textrm{C}^{2\,s+1}(\Omega )} \le \frac{C\bar{C}}{c} \Vert g\Vert _{\textrm{H}^{k}(\Omega )}$ which proves the lemma. $\square$

##### Remark 12

The constant *C* in the previous lemma depends on the choice of *k* and *g*. In particular, we use equivalence of norms between the spaces $\mathcal {H}^k$ and $\textrm{H}^{k}$, Morrey’s inequality to embed $\textrm{C}^{2s+1}$ into $\textrm{H}^{k}$, and the $\textrm{H}^{k}$ norm of *g*. We note, however, that the constant *c* depends on *g* only through $\Vert g\Vert _{\textrm{H}^{k}(\Omega )}$, and this dependence is linear.

We can now prove Proposition 2.7.

##### Proof of Proposition 2.7

We have

$$\begin{aligned} \tau \Delta _n^s u_\tau ^* + u_\tau ^* - g = \tau \left( \Delta _n^s - \Delta _\rho ^{s}\right) u_\tau ^* \end{aligned}$$

on $\Omega _n$. So, letting $w= u_{\tau }^{*}\lfloor _{\Omega _n} - u_{n,\tau }^{g*}$ we can bound

$$\begin{aligned} \tau \Delta _n^s w + w = \tau \left( \Delta _n^s - \Delta _\rho ^{s}\right) u_\tau ^*. \end{aligned}$$

By Lemma 2.13 and Theorem 2.8

$$\begin{aligned} \Vert w\Vert _{\textrm{L}^{2}(\mu _n)} \le \tau \left\| \left( \Delta _n^s - \Delta _\rho ^s\right) u_\tau ^* \right\| _{\textrm{L}^{2}(\mu _n)} \le C\tau \varepsilon \left( \Vert u_\tau ^{*}\Vert _{\textrm{C}^{2s+1}(\Omega )} + 1 \right) \end{aligned}$$

with probability at least $1-Cn^{-\alpha } - Cne^{-cn\varepsilon ^{d+4s}}$. By Lemma 2.14$\Vert u_\tau ^{*}\Vert _{\textrm{C}^{2s+1}(\Omega )}$ can be bounded for all $\tau \in (0,\tau _0)$. This completes the proof of the first inequality.

We can derive the second inequality from the first inequality as follows

$$\begin{aligned} \left\| \Delta _n^{\frac{s}{2}} u_{n,\tau }^{g*} - \Delta _\rho ^{\frac{s}{2}} u_{\tau }^{*}\lfloor _{\Omega _n}\right\| _{\textrm{L}^{2}(\mu _n)}^2&\le 2\left\| \Delta _n^{\frac{s}{2}} u_{n,\tau }^{g*} - \Delta _n^{\frac{s}{2}} u_{\tau }^{*}\right\| _{\textrm{L}^{2}(\mu _n)}^2 \hspace{-11.01pt} + 2 \left\| \Delta _n^{\frac{s}{2}} u_{\tau }^{*} - \Delta _\rho ^{\frac{s}{2}} u_{\tau }^{*}\lfloor _{\Omega _n}\right\| _{\textrm{L}^{2}(\mu _n)}^2 \\&\le 2 \left\| \Delta _n^{s} \left( u_{n,\tau }^{g*} - u_{\tau }^{*}\lfloor _{\Omega _n} \right) \right\| _{\textrm{L}^{2}(\mu _n)} \left\| u_{n,\tau }^{g*} - u_{\tau }^{*}\lfloor _{\Omega _n}\right\| _{\textrm{L}^{2}(\mu _n)} \\&\quad + C\varepsilon ^2\left( \Vert u_\tau ^{*}\Vert _{\textrm{C}^{s+1}(\Omega )} + 1\right) ^2 \end{aligned}$$

with probability at least $1-Cn^{-\alpha }-Cne^{-cn\varepsilon ^{d+4s}}$ where we have used Theorem 2.8 on the second term and the computation

$$\begin{aligned} \left\| \Delta _n^{\frac{s}{2}} u_{n,\tau }^{g*} - \Delta _n^{\frac{s}{2}} u_{\tau }^{*}\right\| _{\textrm{L}^{2}(\mu _n)}^2&= \left\langle \Delta _n^{\frac{s}{2}} (u_{n,\tau }^{g*} - u_{\tau }^{*}), \Delta _n^{\frac{s}{2}}( u_{n,\tau }^{g*} - u_{\tau }^{*}) \right\rangle _{\textrm{L}^{2}(\mu _n)} \\&= \left\langle \Delta _n^s (u_{n,\tau }^{g*} - u_{\tau }^{*}), u_{n,\tau }^{g*} - u_{\tau }^{*} \right\rangle _{\textrm{L}^{2}(\mu _n)} \\&\le \left\| \Delta _n^s (u_{n,\tau }^{g*} - u_{\tau }^{*})\right\| _{\textrm{L}^{2}(\mu _n)} \left\| u_{n,\tau }^{g*} - u_{\tau }^{*} \right\| _{\textrm{L}^{2}(\mu _n)} \end{aligned}$$

on the first term. Comparing the Euler–Lagrange equations we have

$$\begin{aligned} \tau \Delta _n^s \left( u_{n,\tau }^{g*} - u_\tau ^*\lfloor _{\Omega _n} \right) + \left( u_{n,\tau }^{g*} - u_\tau ^* \right) = \tau \left( \Delta _\rho ^s - \Delta _n^s \right) u_\tau ^*. \end{aligned}$$

By Theorem 2.8 we can derive the bound

$$\begin{aligned} \left\| \Delta _n^s \left( u_{n,\tau }^{g*} - u_\tau ^*\lfloor _{\Omega _n} \right) \right\| _{\textrm{L}^{2}(\mu _n)}&\le \frac{1}{\tau } \left\| u_{n,\tau }^{g*} - u_\tau ^*\lfloor _{\Omega _n}\right\| _{\textrm{L}^{2}(\mu _n)} + \left\| \left( \Delta _\rho ^s - \Delta _n^s \right) u_\tau ^* \right\| _{\textrm{L}^{2}(\mu _n)} \\&\le C\varepsilon \left( 1+\Vert u_\tau ^*\Vert _{\textrm{C}^{2s+1}(\Omega )} \right) \end{aligned}$$

with probability at least $1-Cn^{-\alpha }-Cne^{-cn\varepsilon ^{d+4s}}$. Therefore,

$$\begin{aligned} \left\| \Delta _n^{\frac{s}{2}} u_{n,\tau }^{g*} - \Delta _\rho ^{\frac{s}{2}} u_{\tau }^{*}\lfloor _{\Omega _n}\right\| _{\textrm{L}^{2}(\mu _n)}^2 \le C\varepsilon ^2 \left( \tau + (1+\tau ) \Vert u^*_\tau \Vert _{\textrm{C}^{2s+1}(\Omega )}^{2} \right) \end{aligned}$$

with probability at least $1-Cn^{-\alpha }-Cne^{-cn\varepsilon ^{d+4s}}$. If $\tau \le \tau _0$ then we can bound by $C\varepsilon ^2$ as required. $\square$

Putting together Propositions 2.1 and 2.7 proves Theorem 1.1 and Remark 3.

## $\textrm{L}^{2}$ bias estimates

Recalling that the Fréchet derivative of $\mathcal {E}_{\infty ,\tau }^{(g)}$ is

$$\begin{aligned} \frac{1}{2} \nabla _{\text {L}^2(\mu )} \mathcal {E}_{\infty ,\tau }^{(g)}(u) = \tau \Delta _\rho ^s u + u - g \end{aligned}$$

then one can easily check that the following subgradient equality holds

33 $$\begin{aligned} \left\langle \nabla _{\text {L}^2(\mu )} \mathcal {E}_{\infty ,\tau }^{(g)}(w), w-v\right\rangle _{\text {L}^2(\mu )} - \Vert w-v\Vert _{\text {L}^2(\mu )}^2 - \tau \left\| \Delta _\rho ^{\frac{s}{2}} (w-v) \right\| _{\text {L}^2(\mu )}^2 = \mathcal {E}_{\infty ,\tau }^{(g)}(w) - \mathcal {E}_{\infty ,\tau }^{(g)}(v) \end{aligned}$$

for any $v,w \in \text {H}^s(\Omega )$. Since $\nabla _{\text {L}^2(\mu )} \mathcal {E}_{\infty ,\tau }^{(g)}(u_\tau ^*) = 0$ and *g* is sufficiently regular then

$$\begin{aligned} \Vert u_\tau ^* -g\Vert _{\text {L}^2(\mu )}^2 + \tau \left\| \Delta _\rho ^{\frac{s}{2}} (u_\tau ^* - g)\right\| _{\text {L}^2(\mu )}^2&= \mathcal {E}_{\infty ,\tau }^{(g)}(g) - \mathcal {E}_{\infty ,\tau }^{(g)}(u_\tau ^*) \\&= \left\langle \nabla _{\text {L}^2(\mu )} \mathcal {E}_{\infty ,\tau }^{(g)}(g), g-u_\tau ^*\right\rangle _{\text {L}^2(\mu )} - \Vert g-u_\tau ^*\Vert _{\text {L}^2(\mu )}^2 \\&\quad - \tau \left\| \Delta _\rho ^{\frac{s}{2}} (g-u_\tau ^*) \right\| _{\text {L}^2(\mu )}^2 \end{aligned}$$

where for the first equality we let $w=u_\tau ^*$, $v=g$ in (33), and in the second equality we let $w=g$, $v=u_\tau ^*$ in (33). Hence

$$\begin{aligned} \Vert u_\tau ^* -g\Vert _{\text {L}^2(\mu )}^2 + \tau \left\| \Delta _\rho ^{\frac{s}{2}} (u_\tau ^* - g)\right\| _{\text {L}^2(\mu )}^2&= \frac{1}{2}\left\langle \nabla _{\text {L}^2(\mu )} \mathcal {E}_{\infty ,\tau }^{(g)}(g), g-u_\tau ^*\right\rangle _{\text {L}^2(\mu )} \\&\le \frac{1}{2} \left\| \nabla _{\text {L}^2(\mu )} \mathcal {E}_{\infty ,\tau }^{(g)}(g)\right\| _{\text {L}^2(\mu )} \Vert g-u_\tau ^*\Vert _{\text {L}^2(\mu )}. \end{aligned}$$

It follows that

$$\begin{aligned} \left\| u_\tau ^* -g\right\| _{\text {L}^2(\mu )}\le \frac{1}{2} \left\| \nabla _{\text {L}^2(\mu )} \mathcal {E}_{\infty ,\tau }^{(g)}(g)\right\| _{\text {L}^2(\mu )} = \tau \left\| \Delta _\rho ^s g\right\| _{\text {L}^2(\mu )} \end{aligned}$$

and

$$\begin{aligned} \left\| \Delta _\rho ^{\frac{s}{2}} (u_\tau ^* - g)\right\| _{\text {L}^2(\mu )}^2 \le \frac{1}{2} \left\| \nabla _{\text {L}^2(\mu )} \mathcal {E}_{\infty ,\tau }^{(g)}(g)\right\| _{\text {L}^2(\mu )} \left\| \Delta _\rho ^s g\right\| _{\text {L}^2(\mu )} = \frac{\tau }{2} \left\| \Delta _\rho ^s g\right\| _{\text {L}^2(\mu )}^2 \end{aligned}$$

which proves Theorem 1.2 and Remark 5.

## Data Availability

No data was created or analyzed in this study, as such data sharing is not applicable.
